# Specificity and Mechanism of Coronavirus, Rotavirus, and Mammalian Two-Histidine Phosphoesterases That Antagonize Antiviral Innate Immunity

**DOI:** 10.1128/mBio.01781-21

**Published:** 2021-08-10

**Authors:** Abhishek Asthana, Christina Gaughan, Beihua Dong, Susan R. Weiss, Robert H. Silverman

**Affiliations:** a Department of Cancer Biology, Lerner Research Institute, Cleveland Clinic Foundation, Cleveland, Ohio, USA; b Department of Microbiology, Perlman School of Medicine at the University of Pennsylvania, Philadelphia, Pennsylvania, USA; c Penn Center for Research on Coronaviruses and Other Emerging Pathogens, Perlman School of Medicine at the University of Pennsylvania, Philadelphia, Pennsylvania, USA; Stony Brook University

**Keywords:** 2-5A, AKAP7, OAS, RNase L, coronavirus, innate immunity, interferons, rotavirus, two-histidine phosphoesterase

## Abstract

The 2′,5′-oligoadenylate (2-5A)-dependent endoribonuclease, RNase L, is a principal mediator of the interferon (IFN) antiviral response. Therefore, the regulation of cellular levels of 2-5A is a key point of control in antiviral innate immunity. Cellular 2-5A levels are determined by IFN-inducible 2′,5′-oligoadenylate synthetases (OASs) and by enzymes that degrade 2-5A. Importantly, many coronaviruses (CoVs) and rotaviruses encode 2-5A-degrading enzymes, thereby antagonizing RNase L and its antiviral effects. A-kinase-anchoring protein 7 (AKAP7), a mammalian counterpart, could possibly limit tissue damage from excessive or prolonged RNase L activation during viral infections or from self-double-stranded RNAs that activate OAS. We show that these enzymes, members of the two-histidine phosphoesterase (2H-PE) superfamily, constitute a subfamily referred here as 2′,5′-PEs. 2′,5′-PEs from the mouse CoV mouse hepatitis virus (MHV) (NS2), Middle East respiratory syndrome coronavirus (MERS-CoV) (NS4b), group A rotavirus (VP3), and mouse (AKAP7) were investigated for their evolutionary relationships and activities. While there was no activity against 3′,5′-oligoribonucleotides, they all cleaved 2′,5′-oligoadenylates efficiently but with variable activity against other 2′,5′-oligonucleotides. The 2′,5′-PEs are shown to be metal ion-independent enzymes that cleave trimer 2-5A (2′,5′-p_3_A_3_) producing mono- or diadenylates with 2′,3′-cyclic phosphate termini. Our results suggest that the elimination of 2-5A might be the sole function of viral 2′,5′-PEs, thereby promoting viral escape from innate immunity by preventing or limiting the activation of RNase L.

## INTRODUCTION

**FIG 1:**
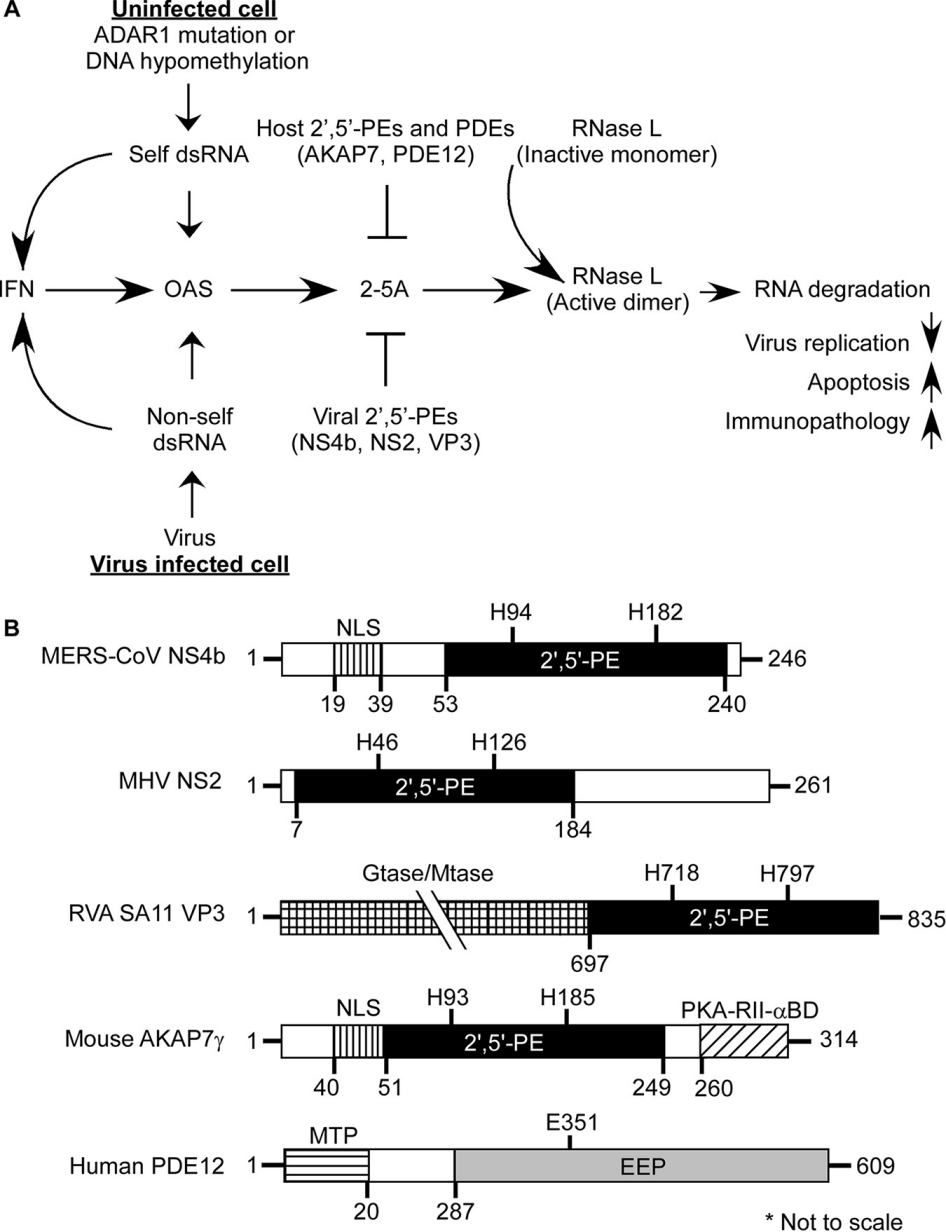
Interplay between cellular responses to viral and host dsRNAs, the OAS-RNase L pathway, and antagonism by 2-5A-degrading enzymes. (A) OAS1–3 are IFN-induced dsRNA sensors. Once activated, they synthesize the antiviral substance 2′,5′-oligoadenylate (2-5A) from ATP. 2-5A binds inactive monomeric RNase L, inducing active RNase L dimers, which in turn degrade viral and host single-stranded RNAs. The balance between 2-5A accumulation by OAS enzymes and its degradation by host and viral enzymes determines cell and virus fate and inflammatory responses. (B) Domain structures of viral and cellular 2′,5′-PEs and human PDE12 (an endonuclease/exonuclease/phosphatase [EEP] family member). Features of full-length MERS-CoV NS4b, MHV NS2, RVA SA11 VP3, and Mus musculus AKAP7γ proteins, including a nuclear localization sequence (NLS) and catalytic 2′,5′-PE domains, are compared (modified from reference 24). Positions of conserved histidines within the catalytic domain of 2′,5′-PEs are shown. PKA-RII-α-BD, binding domain for regulatory subunit II (RII) of cAMP-dependent protein kinase A (27). Guanylyltransferase (Gtase) and methyltransferase (Mtase) domains are also shown (25, 29). The mitochondrial-matrix-targeting peptide (MTP) and the catalytic EEP domain of PDE12 are shown (55). Domains shown are not drawn to scale.

How interferons (IFNs) inhibit viral infections and how viruses antagonize the IFN antiviral response have been investigated for the past few decades but with renewed intensity as a result of the severe acute respiratory syndrome coronavirus 2 (SARS-CoV-2) pandemic (1–4). Mammalian cells often detect and respond to viruses after sensing viral double-stranded RNA (dsRNA), a common viral pathogen-associated molecular pattern (PAMP) that induces type I and type III IFNs (1, 2). These IFNs induce the expression of hundreds of IFN-stimulated genes (ISGs), including numerous antiviral effector proteins (5). Included among the human antiviral proteins encoded by ISGs are 2′,5′-oligoadenylate (2-5A) synthetases 1 to 3 (OAS1–3) consisting of one, two, and three core OAS units, respectively (6–8). However, not all mammalian species express a similar set of homologous OASs, and some, but not all, related OAS like (OASL) proteins lack enzymatic activity (9, 10). Upon binding of and activation by viral dsRNA, OAS1–3 synthesize 2-5A [p_3_(A2′p5′)*_n_*A, where *n* = 2 to >3] from ATP (7). The only known function of 2-5A is the dimerization and activation of RNase L resulting in the degradation of host and viral RNAs and protein synthesis inhibition, apoptosis, and inflammasome activation (11–15) (Fig. 1A). In addition to its antiviral effects, RNase L resulted in cell death in response to mutation of ADAR1 (adenosine deaminase acting on RNA-1) in a cell line or in cells treated with the DNA-demethylating drug 5-aza-cytidine, both of which induce the synthesis of self-dsRNA from repetitive DNA elements in the genome (16–18). Thus, the regulation of 2-5A levels is critical for host cell viability as well as for the control of viral infections and pathogenesis. Yet there are gaps in our knowledge of precisely how levels of 2-5A are established to restrict viral replication and spread by RNase L activation while at the same time minimizing tissue damage to the host.

The regulation of 2-5A degradation is a key point of control in the OAS-RNase L pathway. Previously, we identified several different members of the eukaryotic-viral LigT group of the two-histidine phosphoesterase (2H-PE) superfamily, named for two His-ϕ-Thr/Ser-ϕ motifs (where ϕ is a hydrophobic residue) that degrade 2-5A and therefore function as potent RNase L antagonists (19, 20) (Fig. 1B). Here, we refer to 2H-PE members that degrade 2′,5′-oligoadenylates as 2′,5′-PEs. Other members of the 2H-PE superfamily have different activities, including 2′,3′-cyclic nucleotide phosphodiesterase and 3′,5′-deadenylase activities (19, 21).

The prototype of the 2′,5′-PEs is the mouse coronavirus (CoV) mouse hepatitis virus (MHV) accessory protein NS2 (22). However, predicted or confirmed 2′,5′-PEs are expressed by many betacoronaviruses (embecovirus lineage MHV, human coronavirus [HCoV] OC43, human enteric coronavirus [HECoV], equine coronavirus [ECoV], porcine hemagglutinating encephalomyelitis virus [PHEV], and merbeco lineage Middle East respiratory syndrome CoV [MERS-CoV] and related bat CoVs [BtCoVs]), related toroviruses, and group A and B rotaviruses (20, 23–26). However, the betacoronaviruses SARS-CoV and SARS-CoV-2 lack a 2′,5′-PE. Perhaps as a consequence, SARS-CoV-2 activates, and is inhibited by, the OAS and RNase L pathway (4). In addition, there is also a mammalian 2′,5′-PE, A-kinase-anchoring protein (AKAP7) (also known as AKAP15 or AKAP18) that degrades 2-5A (27). Here, we have expressed, purified, and characterized the 2′,5′-PEs from MHV (NS2), MERS-CoV (NS4b), rotavirus group A (RVA) (VP3 C-terminal domain [VP3-CTD]), and mouse AKAP7 (muAKAP7). We show that NS2 and NS4b are remarkably specific for cleaving 2′,5′-linked oligoadenylates, whereas AKAP7 and VP3-CTD will also cleave other 2′,5′-oligonucleotides. In contrast, all of the viral and mammalian 2′,5-PEs tested lack the ability to cleave 3′,5′-oligoribonucleotides. We further show that these enzymes are metal ion independent and cleave trimer 2-5A (2′,5′-p_3_A_3_), producing mono- and diadenylates with 2′,3′-cyclic phosphoryl termini. Our findings suggest that the sole function of the viral 2′,5′-PEs may be to eliminate 2-5A, allowing some coronaviruses and rotaviruses to evade the antiviral activity of RNase L.

## RESULTS

### Phylogenetic relationship and alignment of viral and cellular 2′,5′-PEs.

To probe the precise molecular mechanism by which 2′,5′-PEs allow some viruses to evade the antiviral effector RNase L, we further investigated MHV NS2, MERS-CoV NS4b (MERS-NS4b), the rotavirus group A (RVA) VP3 C-terminal domain (VP3-CTD), and mouse AKAP7 (muAKAP7) (Fig. 1B). A comparison of the domain organizations of these 2′,5′-PEs shows a related, catalytic domain. Some of these enzymes have additional domains related to intracellular localization, nucleic acid metabolism, or protein binding functions indicative of their cellular compartment-specific or accessory functions (Fig. 1B). For instance, MERS-CoV NS4b and muAKAP7 contain N-terminal nuclear localization signal (NLS) domains (24, 27, 28). VP3 is a multifunctional enzyme that contains N-terminal guanylyltransferase (Gtase) and methyltransferase (Mtase) domains involved in the capping of the 5′ termini of viral mRNAs (25, 29). muAKAP7 also has a carboxy-terminal binding domain for regulatory subunit II (RII) of cyclic AMP (cAMP)-dependent protein kinase A (PKA-RII-α-BD) (27). In addition, MHV NS2 protein has a C-terminal extension of unknown identity or function (Fig. 1B).

**FIG 2:**
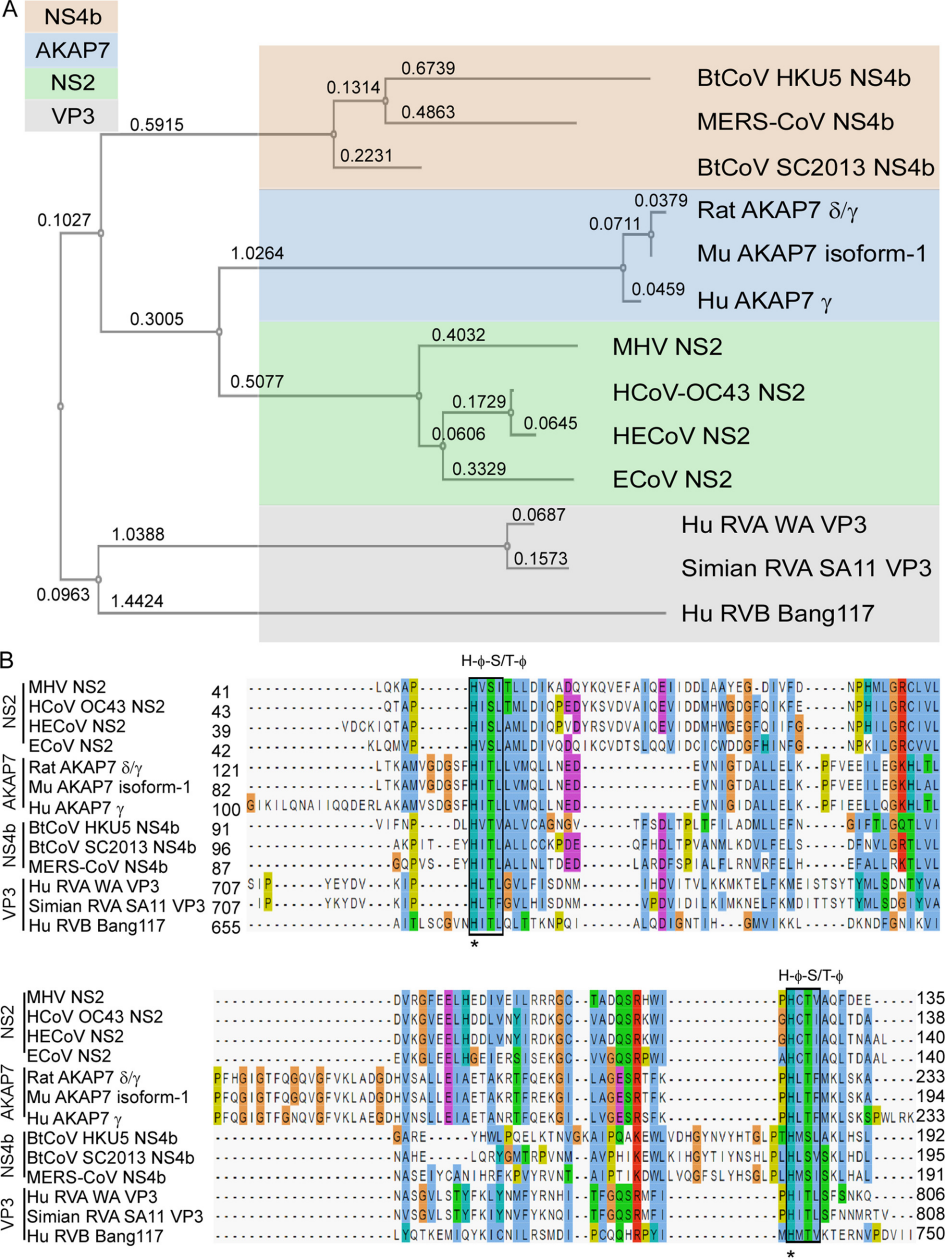
Phylogenetic relationship and sequence alignment of 2′,5′-PEs. (A) Phylogenetic tree based on amino acids from catalytic domains of 2′,5′-PEs. The numbers represent branch lengths. (B) Sequence alignment of amino acids spanning the catalytic domain of 2′,5′-PEs using the MAFFT multiple-sequence alignment program. Catalytic motifs [H-ϕ-(S/T)-ϕ] are indicated above the boxes, where ϕ represents a hydrophobic residue. Numbers represent the start and end of the amino acid sequences used for sequence alignment. Aligned residues are color-coded for conservation according to the Clustal X scheme. Blue, hydrophobic; red, positive charge; magenta, negative charge; green, polar; orange, glycine; yellow, proline; cyan, aromatic; white, unconserved. Hu, human; Mu, mouse; HE, human enteric; E, equine; Bt, bat; CoV, coronavirus; RVA, rotavirus group A; RVB, rotavirus group B.

To determine the phylogenetic relationships between the different 2′,5′-PEs, we constructed a tree for amino acid sequences containing the catalytic domains from coronavirus, rotavirus, and mammalian 2′,5′-PEs (Fig. 2A). 2′,5′-PEs were distributed into two distinct branches on the phylogenetic tree. The VP3 group of proteins clustered into one branch, while the other three groups containing NS2, NS4b, and AKAP7 formed a separate branch (Fig. 2A). Within the VP3 group, RVA and rotavirus group B (RVB) resolved on distinct subbranches. Previously, full-length VP3s from RVA and RVB were also shown as separate distinct branches analogous to two separate clades (clade A and clade B) (30, 31). Interestingly, the NS2 proteins were most closely related to the mammalian AKAP7 catalytic domains and then to the bat coronaviruses (HKU5 and SC2013) and MERS-CoV. The rotavirus VP3 proteins were most distally related to the other 2′,5′-PEs.

Based on the phylogenetic relationship and functional relatedness, we further analyzed the sequence conservation by amino acid alignment. 2H-PE superfamily members are characterized by the presence of two H-ϕ-S/T-ϕ motifs, separated by an average of 80 amino acids (aa) (where ϕ represents a hydrophobic amino acid) (19). The alignment shows that both motifs are highly conserved across all 2′,5′-PE proteins (Fig. 2B, boxes). These motifs form the catalytic core that binds to and cleaves the 2-5A substrate. Consistent with the phylogenetic analysis, sequence analysis revealed that the 2′,5′-PEs clustered into four groups corresponding to NS2, NS4b, AKAP7, and VP3. The two histidines within the conserved motifs were 100% conserved among all the sequences (Fig. 2B, asterisks). Several residues with an intergroup consensus of >50% were identified in the alignment. The amino acid alignment shows several regions of conservation that exist beyond the two conserved catalytic motifs (H-ϕ-S/T-ϕ) (Fig. 2B, shown above the sequence alignment).

Among the sequences in the alignment, AKAP7 proteins of human, rat, and mouse origins shared the highest amino acid identity, ranging between 85 and 97% (88 to 97% similarity) (see Table S1 in the supplemental material). NS2 proteins shared 48 to 92% identity (64 to 94% similarity), NS4b proteins shared 35 to 49% identity (50 to 69% similarity), and VP3 proteins shared 16 to 78% identity (29 to 84% similarity) within their groups. Interestingly, while RVA VP3 proteins shared a high 78% identity (84% similarity) between them, they shared only 16% identity (29% similarity) with the representative of RVB VP3 proteins. The catalytic domains of 2′,5′-PEs have modest intragroup alignment and a relatively lower intergroup alignment. The overall intergroup alignment for the catalytic domains of these proteins showed 10 to 22% identity (19 to 36% similarity) (Table S1). NS2 proteins shared 11 to 16% amino acid identity (24 to 29% similarity) with NS4b, 16 to 22% identity (30 to 36% similarity) with AKAP7, and 16 to 22% identity (26 to 32% similarity) with VP3 proteins. Similarly, the NS4b group of proteins shared 12 to 18% identity (21 to 29% similarity) with AKAP7 and 10 to 19% identity (23 to 34% similarity) with VP3 proteins. AKAP7 and VP3 shared 11 to 20% identity and 19 to 25% similarity between the two groups.

10.1128/mBio.01781-21.7 TABLE S1 Catalytic domain sequence identity and similarity analysis of viral and cellular 2′,5′-PEs. The percent amino acid identity and similarity matrix is based on the alignment in Fig. 2B. 2′,5′-PEs from mammals or mammalian viruses were used for the alignment. Values are calculated using the Sequence Identity and Similarity (SIAS) tool. Matrix values show percent identities (above the diagonal) and similarities (below the diagonal) between the corresponding pairs of proteins. Intragroup identity and similarity values are shaded in gray. Download Table S1, PDF file, 0.2 MB. Copyright © 2021 Asthana et al. 2021 Asthana et al. https://creativecommons.org/licenses/by/4.0/ This content is distributed under the terms of the Creative Commons Attribution 4.0 International license.

### 2′,5′-PEs are specific for 2′,5′-linked phosphodiester bonds and preferably cleave 2′,5′-oligoadenylate.

**FIG 3:**
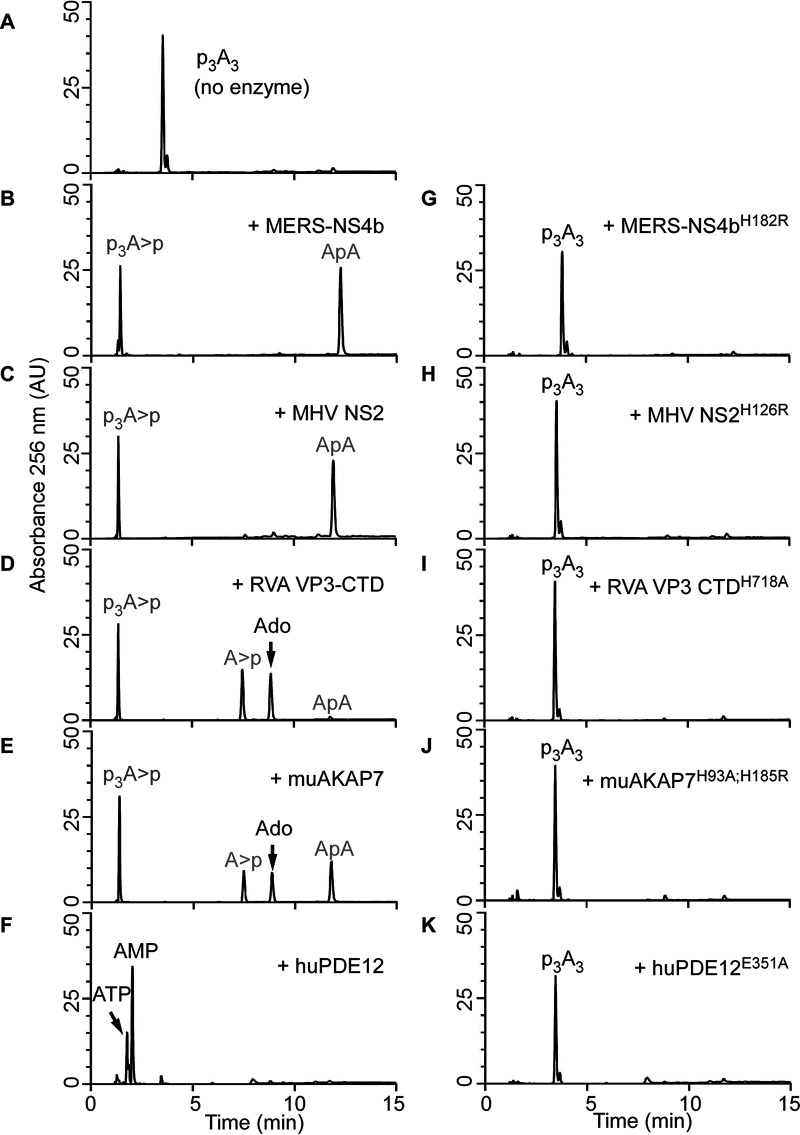
Specific cleavage of trimer 2-5A (2′,5′-p_3_A_3_) by viral and cellular 2′,5′-PEs. (A to F) HPLC analysis of intact 2′,5′-p_3_A_3_ (A), followed by its cleavage with either the viral 2′,5′-PE MERS-NS4b (B), MHV NS2 (C), or RVA VP3-CTD (D) or the host 2′,5′-PE muAKAP7 (E) or human PDE12 (F). Purified 2′,5′-p_3_A_3_ (200 μM) was incubated with 1 μM the indicated proteins at 30°C for 1 h. (G to K) HPLC analysis of catalytically inactive mutants of these enzymes incubated with 2′,5′-p_3_A_3_ under similar conditions for MERS-NS4b^H182R^ (G), MHV NS2^H126R^ (H), RVA VP3-CTD^H718A^ (I), muAKAP7^H93A;H185R^ (J), and human PDE12^E351A^ (K). Experiments performed at least three times produced similar 2′,5′-p_3_A_3_ degradation patterns for each 2′,5′-PE. Arrows indicate elution times of the standards ATP, AMP, and adenosine (Ado). Peaks shown in gray were determined from experiments done in Fig. 5 and 6. AU, arbitrary units.

2′,5′-PEs are members of the LigT family of the 2H-PE superfamily of enzymes, which are involved in RNA processing that can act on diverse substrates (19). Also, members such as MERS-CoV NS4b and muAKAP7 have a functional NLS peptide (24, 27). To determine if there was a wider role for these enzymes beyond cleaving 2-5A, we tested an expanded set of potential substrates. MERS-NS4b, MHV NS2, RVA VP3-CTD, and muAKAP7 proteins were expressed in bacteria and then purified. Also, for comparison, human PDE12 (also known as 2′-PDE), a member of the exonuclease-endonuclease-phosphatase (EEP) family known to cleave 2-5A, was purified (Fig. 1B) (32, 33). The catalytically inactive mutant proteins MERS-NS4b^H182R^, MHV NS2^H126R^, RVA VP3-CTD^H718A^, muAKAP7^H93A;H185R^, and human PDE12^E351A^ served as the negative controls. The purity and identity of trimer 2-5A (2′,5′-p_3_A_3_) were confirmed by high-performance liquid chromatography (HPLC) (Fig. 3A) and mass spectrometry (MS) (see Fig. 5J). Purified 2′,5′-PE proteins were incubated with the 2-5A substrate at 30°C for 1 h, and the 2-5A cleavage products were analyzed by HPLC using a C18 column. All five wild-type proteins cleaved 2-5A as observed by the loss of intact 2-5A and the appearance of peaks for the different cleavage products (Fig. 3B to F). Interestingly, MERS-NS4b (Fig. 3B) and MHV NS2 (Fig. 3C) degraded 2-5A to give two prominent products, whereas RVA VP3-CTD (Fig. 3D) and muAKAP7 (Fig. 3E) gave four products upon the extended degradation of 2-5A, suggesting a difference in either the mechanism or rate of cleavage by these proteins. On the other hand, 2-5A cleavage by human PDE12 (Fig. 3F) results in the formation of two products corresponding to the elution time of the standards ATP and 5′-AMP, as previously described (32). As expected, the 2′,5′-PE catalytically inactive mutant proteins containing a His-to-Arg or His-to-Ala mutation in the conserved histidines did not cleave 2-5A (Fig. 3G to J). Human PDE12 with a Glu-to-Ala mutation at amino acid residue 351 also did not degrade 2-5A, as described previously (34) (Fig. 3K). Importantly, these findings show a different mode of 2-5A cleavage between 2′,5′-PEs, members of the 2H-PE superfamily, and PDE12, a member of the EEP family of phosphodiesterases.

To investigate the expanded substrate specificity of 2′,5′-PEs, we tested the possible cleavage of various 2′-5′- and 3′-5′-linked oligoribonucleotides by HPLC. Purified 2′,5′-PE proteins (1 μM) were incubated with either 2′-5′- or 3′-5′-linked pentaribonucleotide substrates (10 μM) at 30°C for 1 h. Wild-type MERS-NS4b specifically degraded 2′-5′ p5′(rA)_5_ by >99%, while 2′-5′ p5′(rU)_5_, p5′(C)_5_, or p5′(G)_5_ was not degraded (≤4%) (Table S2). The catalytically inactive mutant MERS-NS4b^H182R^ did not degrade any of the tested substrates under similar conditions. Wild-type MHV NS2 also specifically degraded 2′-5′ p5′(rA)_5_ by >99%, while 2′-5′ p5′(rU)_5_, p5′(C)_5_, or p5′(G)_5_ was not degraded (≤7%) (Table S2). Mutant MHV NS2^H126R^ did not degrade any of the tested substrates. These results suggest that MERS-NS4b and MHV NS2 are remarkably specific in degrading 2′-5′-linked oligoadenylate compared to the other substrates. We further tested RVA VP3-CTD, which degraded 2′-5′ p5′(rA)_5_ at >95%, p5′(rU)_5_ at ∼40%, p5′(C)_5_ at ∼90%, and p5′(G)_5_ at ∼6%, while mutant RVA VP3-CTD^H718A^ did not degrade any of the tested substrates (Table S2). Wild-type muAKAP7 degraded 2′-5′ p5′(rA)_5_ at >99%, p5′(rU)_5_ at >95%, p5′(C)_5_ at >95%, and p5′(G)_5_ at >90%, while mutant muAKAP7^H93A;H185R^ did not degrade any of the tested substrates with the exception of 2′-5′ p5′(G)_5_ at ∼40% (Table S2). To ensure that the exclusive cleavage of 2′,5′-oligoadenylates by MERS-NS4b was not due to limiting amounts of enzyme, 10 μM different 2′,5′-linked pentaribonucleotides was incubated with 3-fold-higher concentrations (3 μM) of MERS-NS4b at 30°C for 1 h. Wild-type MERS-NS4b specifically degraded 2′-5′ p5′(rA)_5_ by >99%, while 2′-5′ p5′(rU)_5_, p5′(C)_5_, or p5′(G)_5_ was not degraded (≤6%), suggesting that MERS-NS4b enzymatic activity is specific for the degradation of 2′,5′-oligoadenylates (Table S3). Because MERS-NS4b and MHV NS2 cleaved 2′-5′ p5′(rA)_5_ but not other 2′-5′-linked substrates, we further determined if this was due to a lack of binding to the other substrates. To test this possibility, 10 μM 2′-5′ p5′(rA)_5_ was incubated with 0.2 μM MHV NS2 in the absence or presence of increasing concentrations of 2′-5′ p5′(rU)_5_ at 30°C for 10 min. The amounts of 2′-5′ p5′(rA)_5_ degraded by MHV NS2 in the presence of 0, 3.1, 10, 12.5, 25, 50, and 100 μM were determined by HPLC analysis (Fig. S1). Degradation of 2′-5′ p5′(rA)_5_ by MHV NS2 decreased as the amount of 2′-5′ p5′(rU)_5_ in the reaction mixture increased beyond 10 μM (i.e., ratio of >1:1) (Fig. S1). Our results suggest that 2′-5′ p5′(rU)_5_ was able to bind MHV NS2 and competitively interfere with the ability of MHV NS2 to cleave 2′-5′ p5′(rA)_5_.

10.1128/mBio.01781-21.1 FIG S1 2′,5′-p(A)_5_ degradation by MHV NS2 decreases in the presence of 2′,5′-p(U)_5_. The substrate 2′,5′-p(A)_5_ was incubated with 0.2 μM MHV-NS2 wild-type protein in the absence or presence of the indicated concentrations of 2′,5′-p(U)_5_ at 30°C for 10 min. Samples were processed and analyzed by HPLC. 2′,5′-p(A)_5_ incubated under similar conditions in the absence of MHV-NS2 and 2′,5′-p(U)_5_ served as the nondegraded control. Experiments were performed three times (*n* = 3), and bars represent the standard errors of the means. Statistical significance was calculated using an unpaired *t* test (*n* = 3) (*, *P* value of <0.05; **, *P* value of <0.005; ***, *P* value of <0.001; ns, not significant) in GraphPad Prism (9.0.0) software. Download FIG S1, PDF file, 0.2 MB. Copyright © 2021 Asthana et al. 2021 Asthana et al. https://creativecommons.org/licenses/by/4.0/ This content is distributed under the terms of the Creative Commons Attribution 4.0 International license.

10.1128/mBio.01781-21.8 TABLE S2 MERS-NS4b-, MHV NS2-, RVA VP3-CTD-, and muAKAP7-mediated degradation of 5′-phosphorylated 2′-5′- or 3′-5′-linked pentaribonucleotide substrates. A total of 10 μM the indicated substrate was incubated with 1 μM wild-type or mutant 2′,5′-PE for 1 h at 30°C. Percent substrate degradation was calculated by measuring the areas under the peaks in the HPLC chromatograms. Results were reproduced in at least two independent experiments. Download Table S2, PDF file, 0.2 MB. Copyright © 2021 Asthana et al. 2021 Asthana et al. https://creativecommons.org/licenses/by/4.0/ This content is distributed under the terms of the Creative Commons Attribution 4.0 International license.

10.1128/mBio.01781-21.9 TABLE S3 MERS-NS4b-mediated degradation of 5′-phosphorylated 2′-5′- or 3′-5′-linked pentaribonucleotide substrates. A total of 10 μM the indicated substrate was incubated with 3 μM wild-type or mutant MERS-NS4b for 1 h at 30°C. Percent substrate degradation was calculated by measuring the areas under the peaks in the HPLC chromatograms. Results were reproduced in two independent experiments. Download Table S3, PDF file, 0.2 MB. Copyright © 2021 Asthana et al. 2021 Asthana et al. https://creativecommons.org/licenses/by/4.0/ This content is distributed under the terms of the Creative Commons Attribution 4.0 International license.

We next tested the degradation activity of 2′,5′-PEs against 3′-5′-linked p5′(rA)_5_, p5′(rU)_5_, and p5′(C)_5_. One micromolar enzyme was incubated with 10 μM the substrate at 30°C for 1 h. Wild-type MERS-NS4b and its mutant MERS-NS4b^H182R^, wild-type MHV NS2 and its mutant NS2^H126R^, RVA VP3-CTD and its mutant RVA VP3-CTD^H718A^, and wild-type muAKAP7 and its mutant muAKAP7^H93A;H185R^ (Table S2) did not degrade the 3′-5′-linked substrates 3′-5′ p5′(A)_5_, 3′-5′ p5′(U)_5_, and 3′-5′ p5′(C)_5_. [We were unable to obtain 3′-5′ p5′(G)_5_ because of repeated failures of its chemical synthesis and/or purification; therefore, this oligonucleotide could not be tested]. Our results suggest that all of the 2′,5′-PEs examined are highly specific for cleaving 2′,5′-linked oligoribonucleotides. Among the 2′,5′-linked substrates, MERS-NS4b and MHV NS2 are specific for cleaving 2′-5′-oligoadenylate, whereas RVA VP3-CTD cleaved, in order, 2′-5′ pA5 > pC5 > pU5 ≫ pG5, and muAKAP7 cleaved all of the 2′,5′-linked pentanucleotides with similar efficacy.

Based on the differential enzymatic activities of these 2′,5′-PEs in degrading different types of 2′,5′-linked phosphodiester substrates, we tested if they could degrade 2′,3′-cyclic GMP-AMP (cGAMP). cGAMP is a cyclic dinucleotide secondary messenger with mixed phosphodiester linkages between 2′-OH of GMP to the 5′-phosphate of AMP and 3′-OH of AMP to the 5′-phosphate of GMP, synthesized by cyclic GMP-AMP synthase (cGAS) in response to cytoplasmic double-stranded DNA (dsDNA) (35). cGAMP was incubated either with or without wild-type and mutant 2′,5′-PEs at 30°C for 1 h and analyzed by HPLC. Wild-type MERS-NS4b, MHV NS2, RVA VP3-CTD, and muAKAP7 did not degrade 2′,3′-cGAMP, whereas they did degrade 2′,5′-p_3_A_3_ (which served as a positive control) under similar conditions (Table S4). Catalytic mutants of 2′,5′-PEs tested did not degrade 2′,3′-cGAMP or 2′,5′-p_3_A_3_ under similar conditions. The results suggest that 2′,5′-PEs are capable of cleaving 2′,5′-phosphodiester bonds in linear homoribonucleotides but not in the cyclic mixed-phosphodiester-linked 2′,3′-cGAMP.

10.1128/mBio.01781-21.10 TABLE S4 Incubations of 2′,5′-PEs with 2′,3′-cGAMP and 2′,5′-p_3_A_3_. A total of 10 μM the indicated substrate was incubated with 1 μM wild-type or mutant 2′,5′-PEs for 1 h at 30°C. The substrates without enzyme incubated under similar conditions were used as undegraded controls. Percent substrate degradation was calculated by measuring the areas under the peaks in the HPLC chromatograms. Results were reproduced in two independent experiments. Download Table S4, PDF file, 0.2 MB. Copyright © 2021 Asthana et al. 2021 Asthana et al. https://creativecommons.org/licenses/by/4.0/ This content is distributed under the terms of the Creative Commons Attribution 4.0 International license.

### 2′,5′-PEs exhibit metal ion-independent phosphodiesterase activity.

**FIG 4:**
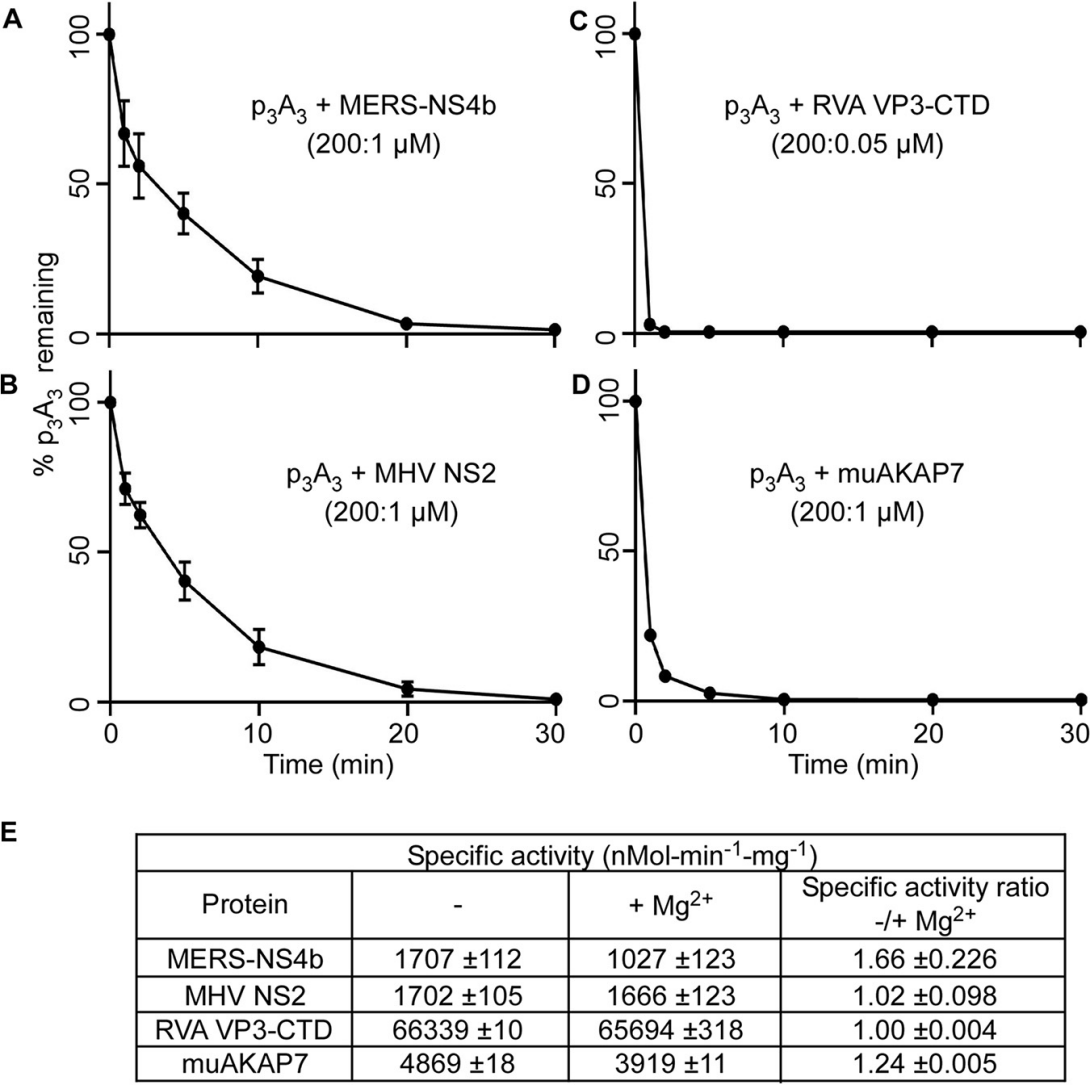
Influence of Mg^2+^ ions on the degradation of 2′,5′-p_3_A_3_ by 2′,5′-PEs. (A to D) Purified 2′,5′-p_3_A_3_ was incubated with the indicated 2′,5′-PEs in time course experiments. Data were obtained by incubating 2′,5′-p_3_A_3_ (200 μM) with MERS-NS4b (1 μM) (A), MHV NS2 (1 μM) (B), RVA VP3-CTD (0.05 μM) (C), and muAKAP7 (1 μM) (D) at 30°C. Samples were collected at 1, 2, 5, 10, 20, and 30 min, and reactions were stopped. The percentages of uncleaved 2′,5′-p_3_A_3_ remaining at the indicated times were determined by calculating the areas under the peaks on the HPLC chromatograms. (E) Table showing the specific activity of 2′,5′-PEs in the absence and presence of 10 mM MgCl_2_. Activity is expressed as the amount of product released from the substrate in nanomoles per minute per milligram of the protein at 30°C during a 5-min reaction time. Experiments were performed in triplicate (*n* = 3), and the standard errors of the means were calculated.

Metal ion dependency was evaluated by performing assays in the presence of either EDTA or magnesium. In the presence of EDTA, without added magnesium, 1 μM MERS-NS4b (Fig. 4A) or MHV NS2 (Fig. 4B) degraded >90% of 2-5A in ∼20 min, whereas 0.05 μM RVA VP3-CTD (Fig. 4C) and 1 μM muAKAP7 (Fig. 4D) degraded >90% of the 2-5A within 5 min. Relative rates of 2-5A degradation by RVA VP3-CTD ≫ muAKAP7 > MHV NS2 = MERS-NS4b were observed. Based on the specific activities, the ratio of fold activities of RVA VP3-CTD: muAKAP7: MERS-NS4b: MHV NS2 was 38.9:2.9:1:1. It is noteworthy that many mammalian cell types have a total cellular Mg^2+^ concentration of between 17 and 20 mM, of which only 5 to 22% may be free depending on the cellular compartment (36). We determined the specific activities of the 2′,5′-PEs for degrading 2-5A in the absence and presence of 10 mM MgCl_2_ at 5 min (Fig. 4E). The addition of Mg^2+^ ions decreased the specific activity of MERS-NS4b to ∼0.6-fold and that of muAKAP7 to ∼0.8-fold. The activities of MHV NS2 and RVA VP3-CTD showed a negligible decrease to 0.97-fold and 0.99-fold in the presence of Mg^2+^ ions, respectively. Our results suggest that the 2′,5′-PE activity of these proteins is independent of Mg^2+^ ions and that its presence either slightly decreases or has no effect on the activity of these enzymes.

### 2′,5′-PEs cleave 2′,5′-linked oligoadenylate leaving products with cyclic 2′,3′-phosphoryl termini.

**FIG 5:**
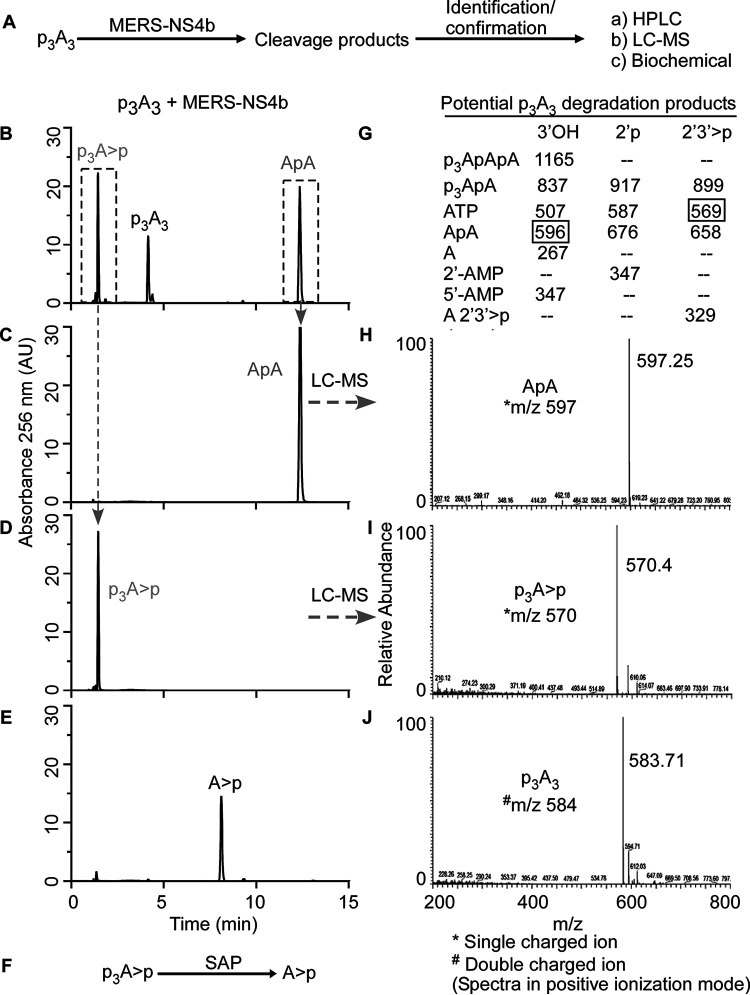
MERS-NS4b cleaves 2′,5′-p_3_A_3_ and catalyzes the formation of 2′,3′-cyclic phosphate products. (A) Schematic illustration of the strategy to identify the cleavage mechanism and degradation products of 2′,5′-p_3_A_3_ by MERS-NS4b. (B) Chromatogram of partially degraded 2′,5′-p_3_A_3_ and cleavage products formed by MERS-NS4b. A total of 200 μM 2′,5′-p_3_A_3_ was incubated with 1 μM MERS-NS4b at 30°C for 10 min. (C) HPLC chromatogram of the collected peak (corresponding to ApA). (D) HPLC chromatogram of the collected peak (corresponding to p_3_A>p). (E) HPLC analysis of the dephosphorylated product (A>p) of the peak collected in panel D (p_3_A>p). (F) Schematic illustration showing that shrimp alkaline phosphatase (SAP)-mediated p_3_A>p dephosphorylation at that 5′ end forms A>p. (G) Expected masses of potential 2′,5′-p_3_A_3_ degradation products containing 3′-OH, 2′p, or 2′,3′>p groups. The box shows masses of actual cleavage products identified by mass spectrometry. (H to J) Mass spectrometry analysis showing the *m/z* (mass-to-charge ratio) of ApA → peak fraction collected in panel C (H), p_3_A>p → peak collected in panel D (I), and intact 2′,5′-p_3_A_3_ (J) Peaks shown in gray were identified in the subsequent experiments.

**FIG 6:**
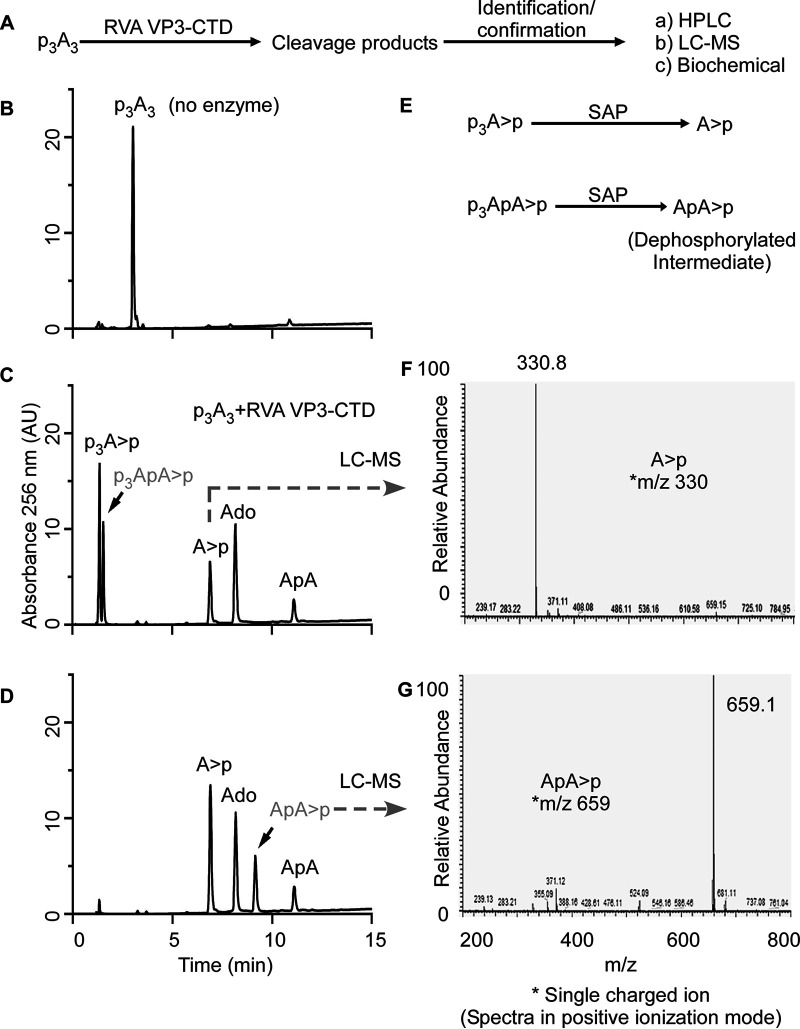
2′,5′-p_3_A_3_ cleavage by RVA VP3-CTD proceeds via the formation of p_3_ApA>p and ApA intermediates. (A) Schematic illustration of the strategy to identify the cleavage mechanism, intermediates, and end products of 2′,5′-p_3_A_3_ cleavage by RVA VP3-CTD. (B) Chromatogram of intact p_3_A_3_. (C) HPLC chromatogram of cleavage products formed by the degradation of 200 μM 2′,5′-p_3_A_3_ incubated with 0.05 μM RVA VP3-CTD for 20 min at 30°C. Peaks identified based on the elution times of known standards are marked. (D) HPLC analysis of the dephosphorylated products from the reaction in panel C. Shrimp alkaline phosphatase (SAP) treatment dephosphorylates 5′-phosphates. The peaks indicated were collected and identified by LC-MS/MS analysis. (E) Schematic illustration showing the dephosphorylation of potential intermediates at the 5′ end using SAP. (F and G) Mass spectrometry analysis showing the *m/z* (mass-to-charge ratio) of A>p (F) and ApA>p (G). Peaks shown in gray were identified in the subsequent experiments.

Differences in the 2-5A cleavage products as determined by HPLC (Fig. 3) suggested that viral and mammalian 2′,5′-PEs cleave 2-5A via a different mechanism than that of human PDE12, which degrades 2-5A to produce ATP and AMP (32). Among 2′,5′-PEs, MERS-NS4b and MHV NS2 degraded 2-5A to give two cleavage products, whereas RVA VP3-CTD and muAKAP7 gave four cleavage products. Therefore, we decided to determine the precise sites of cleavage in 2-5A by 2′,5′-PEs. 2-5A was partially digested with MERS-NS4b (Fig. 5) or RVA VP3-CTD (Fig. 6), followed by the collection of individual peak fractions of cleavage products. Cleavage products were subsequently identified and confirmed by HPLC analysis (comparing elution times with known standards), identification by the mass/charge ratio (*m/z* ratio) (liquid chromatography-tandem mass spectrometry [LC-MS/MS] analysis of the collected peaks), or biochemical analysis (by 5′-dephosphorylation) (Fig. 5A and Fig. 6A).

Partial digestion of 2-5A by MERS-NS4b was confirmed by HPLC analysis of the samples using a C18 column (Fig. 5B) and comparing it with the chromatogram of intact 2-5A (Fig. 3A). The 2-5A partially digested by MERS-NS4b was run on a Dionex DNAPac PA-100 column in an ammonium bicarbonate volatile buffer system as described in Materials and Methods. Individual peaks were collected and processed for mass spectrometry analysis. Individually collected peaks were also rerun on a C18 column to confirm the purity and matched elution times of the collected peaks before performing LC-MS/MS analysis (Fig. 5C and D). Mass spectrometric analysis of the peaks revealed *m/z* ratios of 597.25 (Fig. 5H) and 570.4 (Fig. 5I). The *m/z* ratios of 597.25 and 570.4 were compared to the masses of potential 2-5A degradation/intermediate products (Fig. 5G) and were found to correspond to ApA and p_3_A>p (where “>p” represents a 2′,3′-cyclic phosphate), respectively. Intact 2-5A gave an *m/z* ratio of 584 for the doubly charged ion (Fig. 5J). Moreover, the collected peak of p_3_A>p (shown in Fig. 5D) was subjected to shrimp alkaline phosphatase (SAP)-mediated 5′ dephosphorylation, which results in the peak corresponding to A>p (Fig. 5E and F). This experiment suggested that MERS-NS4b degrades 2-5A to produce a 5′ product with a 2′,3′-cyclic phosphate terminus in the form of p_3_A>p and a 3′ product of ApA. To test if p_3_A>p and ApA are end products of the reaction, we subjected 2-5A to extended degradation by MERS-NS4b and monitored the area under the peak corresponding to ApA at 0 h, 1 h, 4 h, and 24 h (Fig. S2A to D and I). After the ApA peak appears (at 1 h), its amount remained unchanged up to 24 h. Also, 2′-5′-linked 5′pApA incubated with MERS-NS4b did not result in any degradation (Fig. S2E to I). These results suggest that MERS-NS4b does not cleave diadenylates into smaller products irrespective of the 5′-monophosphorylation status under the given experimental conditions.

10.1128/mBio.01781-21.2 FIG S2 MERS-NS4b degrades 2′,5′-p_3_A_3_ but not 2′,5′-linked ApA or pApA. (A and B) The substrate 2′,5′-p_3_A_3_ (A) was degraded in the presence of NS4b into p_3_A>p and ApA (B). (B to D) No appreciable decrease in the amounts of ApA (area under the peak) after incubation for 1, 4, and 24 h, respectively. (E to H) HPLC chromatograms of the substrate pApA in the presence of NS4b at 0, 1, 4, and 24 h. (I) Table showing the amount of ApA or pApA degraded by NS4b as a function of time. Download FIG S2, PDF file, 0.2 MB. Copyright © 2021 Asthana et al. 2021 Asthana et al. https://creativecommons.org/licenses/by/4.0/ This content is distributed under the terms of the Creative Commons Attribution 4.0 International license.

With a similar approach, we designed an experiment to elucidate the cleavage products and intermediates formed upon 2-5A degradation by RVA VP3-CTD (Fig. 6A). Because RVA VP3-CTD has high specific activity against 2-5A (Fig. 4), 2-5A was incubated with decreased protein concentrations and times of incubation to capture any possible intermediates and degradation products of 2-5A (Fig. 6B and C). 2-5A cleaved by VP3-CTD forms products, which, based on the elution times of the known standards and compounds, were identified as p_3_A>p, A>p, ApA, adenosine, and an unknown intermediate (shown in gray) (Fig. 6C). Based on the potential degradation intermediates, we speculated that the unknown intermediate was p_3_ApA>p. To test this possibility, a part of the sample reaction mixture with 2-5A cleavage products (obtained from the sample used in Fig. 6C) was subsequently treated with SAP to remove 5′ phosphorylation from the cleavage products (if any), which would result in the formation of A>p and ApA>p from p_3_A>p and p_3_ApA>p, respectively (Fig. 6E). The dephosphorylated sample was analyzed by running it on a C18 column. After SAP treatment, the amounts of adenosine and ApA remained constant compared to those before (Fig. 6C) and after (Fig. 6D) dephosphorylation, as calculated by integrating the areas under the peaks of the HPLC chromatograms. However, the total area under the peak corresponding to A>p increased, suggesting that A>p was formed as a result of the dephosphorylation of p_3_A>p (Fig. 6C and D). Importantly, a new peak (possibly ApA>p) appears, which is formed by the dephosphorylation of an unknown intermediate (Fig. 6D, shown in gray). The dephosphorylated samples were run on a Dionex DNAPac PA-100 column in an ammonium bicarbonate volatile buffer system as described in Materials and Methods. Individual peak fractions were collected and processed for mass spectrometry analysis. Collected peaks were rerun on a C18 column to confirm the purity and match the elution time of the collected peak with those of A>p and the “dephosphorylated intermediate” (Fig. 6D) before performing LC-MS/MS. Mass spectrometric analysis of the peaks revealed *m/z* ratios of 330, corresponding to A>p (Fig. 6F), and 659.1, which corresponds to ApA>p (Fig. 6G). This experiment suggests that trimer 2-5A (2′,5′p_3_A_3_) degradation by RVA VP3-CTD proceeds via the formation of p_3_ApA>p and ApA intermediates. RVA VP3-CTD degrades p_3_ApA>p to form p_3_A>p (5′ product) and A>p (3′ product), whereas diadenylate (ApA) is further degraded to yield A>p (5′ product) and adenosine (3′ product). The complete degradation of p_3_A_3_ by RVA VP3-CTD results in the formation of p_3_A>p, A>p, and adenosine as end products (Fig. 3D). Furthermore, the preferred site of p_3_A_3_ cleavage by RVA VP3-CTD was investigated in a time course experiment. RVA VP3-CTD (0.05 μM) was incubated with the p_3_A_3_ (200 μM) substrate at 30°C, and samples were collected at different time points. The substrate or product peaks at each time point were analyzed by calculating the percentages of the areas under the peaks of the HPLC chromatograms (Fig. S3) and tabulated (Fig. S4A). The analysis revealed that the majority of p_3_A_3_ is cleaved by RVA VP3-CTD to produce p_3_ApA>p (5′ product) and adenosine (3′ product). The intermediate species (p_3_ApA>p) is subsequently cleaved to produce p_3_A>p (5′ product) and A>p (3′ product). A minor fraction of p_3_A_3_ is cleaved by RVA VP3-CTD to produce p_3_A>p (5′ product) and ApA (3′ product). The diadenylate intermediate (ApA) is subsequently cleaved into A>p (5′ product) and adenosine (3′ product) (Fig. S4B), which is apparent from incubations at a higher concentration of RVA VP3-CTD (Fig. 3D). Moreover, the degradation pattern of the two diadenylate intermediates reveals that a triphosphorylated intermediate (p_3_ApA>p) is readily cleaved by RVA VP3-CTD, whereas ApA cleavage is slow, suggesting that 5′-triphosphorylated molecules are preferred over nonphosphorylated substrates (Fig. S3 and S4). In a separate experiment, 10 μM 2′-5′-linked 5′p(A)_2_ was incubated with 1 μM VP3-CTD at 30°C for 1 h. The results confirmed the formation of cleavage products corresponding to pA>p (5′ product) and adenosine (3′ product) (Fig. S5). Moreover, in another time course experiment, muAKAP7 cleaved p_3_A_3_ to produce p_3_A>p (5′ product) and ApA (3′ product) (Fig. S6). The ApA intermediate was further cleaved to form A>p and adenosine. Interestingly, unlike RVA VP3-CTD, muAKAP7-mediated cleavage of p_3_A_3_ does not form a p_3_ApA>p intermediate.

10.1128/mBio.01781-21.3 FIG S3 Time course of 2′,5′-p_3_A_3_ cleavage by RVA VP3-CTD. Purified 2′,5′-p_3_A_3_ (200 μM) was incubated with RVA VP3-CTD (0.05 μM) at 30°C. Samples were collected at 0 min (A), 2 min (B), 5 min (C), 10 min (D), and 30 min (E) and analyzed by HPLC. The percentages of the substrate or products at the indicated times were determined by calculating the areas under the peaks on the HPLC chromatograms. The right-hand side shows major and minor reactions proceeding at the indicated time points deduced from HPLC chromatogram analysis. Download FIG S3, PDF file, 0.2 MB. Copyright © 2021 Asthana et al. 2021 Asthana et al. https://creativecommons.org/licenses/by/4.0/ This content is distributed under the terms of the Creative Commons Attribution 4.0 International license.

10.1128/mBio.01781-21.4 FIG S4 Mechanism of 2′,5′-p_3_A_3_ cleavage by RVA VP3-CTD. (A) The percentages of the substrate or the products at the indicated times were determined by calculating the areas under the peaks on the HPLC chromatograms obtained in the experiment from Fig. S3 in the supplemental material. (B) Summary of major and minor reactions involved in the cleavage of 2′,5′-p_3_A_3_ by VP3-CTD. The minor reaction cleavage of ApA to A>p and Ado is inferred from incubations performed at a 20-fold-higher concentration of RVA VP3-CTD, 1 μM (Fig. 3D). Download FIG S4, PDF file, 0.2 MB. Copyright © 2021 Asthana et al. 2021 Asthana et al. https://creativecommons.org/licenses/by/4.0/ This content is distributed under the terms of the Creative Commons Attribution 4.0 International license.

10.1128/mBio.01781-21.5 FIG S5 RVA VP3-CTD degrades 2′,5′-linked diadenylate. The substrate 2′,5′-pApA (A) was incubated with 1 μM either wild-type RVA VP3-CTD (B) or its mutant RVA VP3 CTD^H718A^ (C) at 30°C for 1 h. Samples were processed and analyzed by HPLC. The baseline buffer signal was subtracted from the samples. Download FIG S5, PDF file, 0.2 MB. Copyright © 2021 Asthana et al. 2021 Asthana et al. https://creativecommons.org/licenses/by/4.0/ This content is distributed under the terms of the Creative Commons Attribution 4.0 International license.

10.1128/mBio.01781-21.6 FIG S6 Time course of 2′,5′-p_3_A_3_ cleavage by muAKAP7. Purified 2′,5′-p_3_A_3_ (200 μM) was incubated with muAKAP7 (1 μM) at 30°C. (A to E) Samples were collected at 0 min (A), 2 min (B), 10 min (C), 30 min (D), and 60 min (E) and analyzed by HPLC. The peaks were identified by comparison to the elution times of known standards. The percentages of the substrate or products at the indicated times were determined by calculating the areas under the peaks on the HPLC chromatograms. (F) Schematics showing the cleavage of 2′,5′-p_3_A_3_ by muAKAP7. The reaction intermediate is shown in gray. Download FIG S6, PDF file, 0.2 MB. Copyright © 2021 Asthana et al. 2021 Asthana et al. https://creativecommons.org/licenses/by/4.0/ This content is distributed under the terms of the Creative Commons Attribution 4.0 International license.

**FIG 7:**
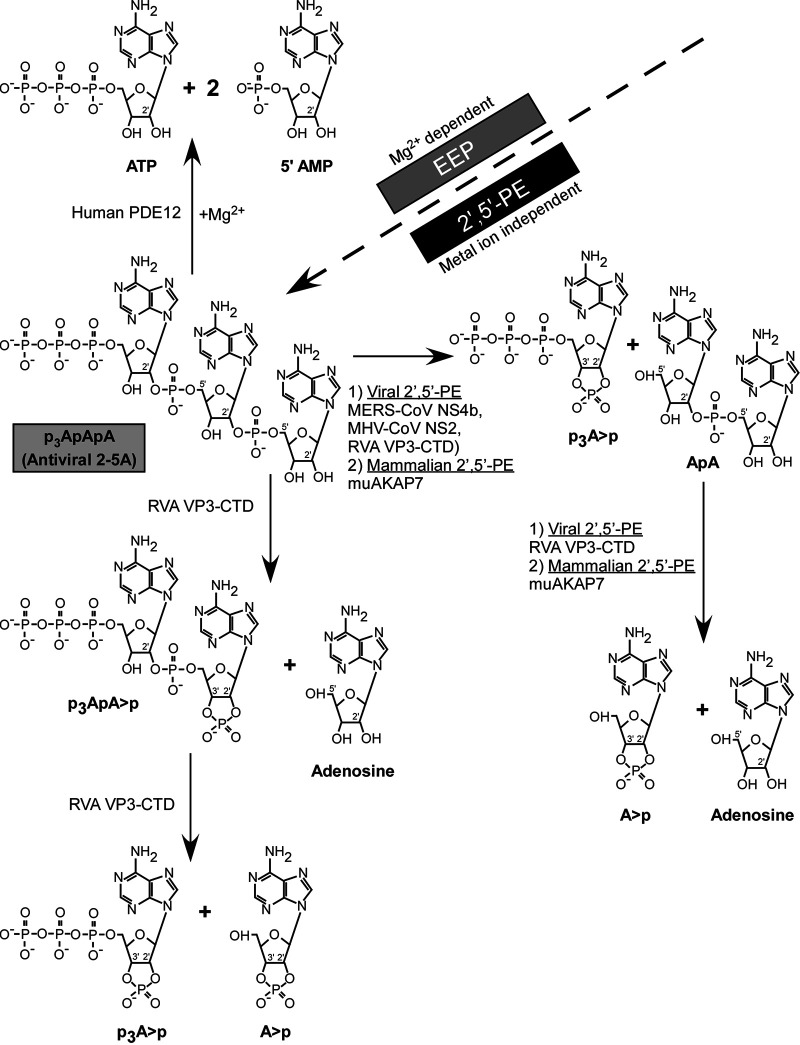
Mechanism of 2′-5′-p_3_A_3_ degradation by the 2′,5′-PE (subfamily of the 2H-PE superfamily) and EEP (endonuclease/exonuclease/phosphatase) families. MERS-CoV NS4b, MHV-CoV NS2, RVA VP3, and mammalian mouse AKAP7 from the 2′,5′-PE subfamily cleave 2′,5′-p_3_A_3_ and leave 2′,3′ >p groups on the 5′ products, while human PDE12, an EEP family member, degrades 2′,5′-p_3_A_3_ to yield ATP and AMP.

The overall mechanisms and differences in the degradation of 2-5A by representative EEP (PDE12) and 2′,5′-PE family members are summarized in Fig. 7. Human PDE12 degrades trimer 2-5A into ATP and 2(5′-AMP)s in the presence of Mg^2+^ ions, as has been reported previously (32). On the other hand, mammalian and viral 2′,5′-PEs act in a metal ion-independent way, degrading 2-5A to form 5′ products with 2′,3′-cyclic phosphates. All 2′,5′-PEs quickly cleave active antiviral 2-5A into inactive molecules; that is, the products are not capable of activating RNase L because of a requirement for at least 3 adenylyl residues (37). MERS-NS4b and MHV NS2 degrade trimer 2-5A to form p_3_A>p and ApA. RVA VP3-CTD and muAKAP7 further cleave ApA to form A>p and adenosine as products. In addition to the above-mentioned degradation intermediates and products of 2-5A, RVA VP3-CTD also produced p_3_ApA>p as an intermediate, suggesting that it is a 2′,5′-specific endoribonucleolytic phosphodiesterase (Fig. 6 and 7).

## DISCUSSION

### Cleavage specificity and mechanism of 2′,5′-PEs.

The 2′,5′-PEs studied here exclusively cleaved 2′,5′- and not 3′,5′-phosphodiester bonds. There was also a strong preference for the cleavage of 2′,5′-oligoadenylates by NS2, NS4b, and, to a lesser extent, VP3-CTD, whereas AKAP7 had similar activities against the different 2′,5′-linked pentamers of A, U, C, and G. Therefore, although AKAP7 and VP3-CTD are not the most closely related 2′,5′-PEs, they can both cleave 2′,5′-oligoribonucleotides other than 2-5A (Fig. 2A; see also Table S2 in the supplemental material). Interestingly, OASs are 2′-nucleotidyltransferases that not only use ATP as the substrate but also can produce diverse molecules with 2′,5′ linkages. NAD^+^, tRNAs, A5′p_4_5′A, and mono- and poly-ADP-ribose are acceptors for the addition of 2′,5′-AMP residues from ATP by OAS. Also, OAS can add other 2′-terminal ribo- and deoxynucleotide monophosphates to 2-5A (38–41). However, which, if any, of these alternative 2-5A-like molecules can be cleaved by 2′,5′-PEs remains to be determined. cGAMP, a cyclic dinucleotide that activates STING, has one 2′,5′ linkage and one 3′,5′ linkage, but it is not cleaved by the 2′,5′-PEs examined here (Table S4). VP3 was phylogenetically distal and has the most distinct mechanism of 2-5A cleavage compared to all of the tested 2′,5′-PEs. It is also interesting to note that the two coronavirus 2′,5′-PEs (NS4b and NS2) are less closely related than NS2 is to the host enzyme AKAP7 (Fig. 2A and Table S1). Our results suggest that the main, and perhaps only, function of these activities is to degrade 2-5A, thus preventing RNase L activation and viral escape or, in the case of AKAP7, reducing cell and tissue damage from RNase L activity. These 2′,5′-PEs are also metal ion-independent enzymes, as is RNase L (42).

The viral and mammalian 2′,5′-PEs produce cleavage products from trimer 2-5A (2′,5′-p_3_A_3_) with cyclic 2′,3′-phosphoryl groups and not 2′,3′-OH termini. These conclusions are based on analysis of 2-5A cleavage products by two types of HPLC columns (Dionex and C18) and, importantly, by mass spectrometry. In contrast, our previous studies based on a more limited analysis of the 2-5A cleavage products by one type of HPLC column (Dionex) misidentified these cleavage products of NS2, VP3-CTD, and AKAP7 as AMP and ATP (22, 25, 27).

Interestingly, mammalian and viral 2′,5′-PEs have activities highly similar to those of an invertebrate 2H-PE present in the oyster Crassostrea gigas (43). The oyster enzyme has sequence similarity to AKAP7, is metal ion independent, cleaves 2′,5′- but not 3′,5′-linked oligonucleotides, and leaves cyclic 2′,3′-phosphate and 5′-OH termini on its products. It also degraded triphosphorylated 2-5A oligomers with multifold efficiency compared to the corresponding nonphosphorylated core 2-5A oligomers. Similarly, we observed that RVA VP3-CTD degrades 5′-triphosphorylated diadenylate with 2′,3′-cyclic phosphoryl termini (p3ApA>p) preferentially compared to the nonphosphorylated diadenylate (ApA) core molecule (Fig. S3 and S4). However, the function and role of 2-5A-cleaving enzymes in invertebrates are still unknown.

It is also unknown if the 2′,3′-cyclic phosphates on 2-5A breakdown products generated by 2′,5′-PEs have cell signaling functions. However, small self-RNAs with 2′,3′-cyclic phosphate termini (generated by RNase L) induced IFN-β expression through RIG-I, MDA5, and MAVS (44). Additionally, RNase L-cleaved RNA with 2′,3′-cyclic phosphates stimulated the NLRP3 inflammasome, leading to interleukin-1β (IL-1β) secretion (15). Also, during Staphylococcus aureus infections, RNase T2 cleaves single-stranded RNA (ssRNA), producing purine-2′,3′-cyclic phosphate-terminated oligonucleotides sensed by Toll-like receptor 8 (TLR8) (45). The mammalian enzyme USB1, another 2H-PE, also produces 2′,3′-phosphoryl termini during the deadenylation of U6 snRNA, but it clearly differs from the 2′,5′-PEs because it cleaves 3′,5′-phosphodiester bonds (21).

### 2-5A catabolism during IFN-induced cellular responses to viral and host dsRNA.

The ability of viruses to evade or antagonize the IFN response contributes to viral tropism and disease pathogenesis. Accordingly, viruses have evolved or acquired diverse strategies to overcome inhibition by type I and type III IFNs, both of which induce the transcription of OAS genes (1, 2, 46). However, the precise cellular and molecular mechanisms by which viruses impede tissue-specific host defenses leading to virus-induced pathology continue to be investigated. With regard to the OAS-RNase L pathway, it is the balance between 2-5A anabolic (OAS) and catabolic (e.g., 2′,5′-PEs and PDE12) (20) activities that determines whether virus replication is blocked by RNase L. For instance, RNase L fails to inhibit the coronaviruses MHV and MERS-CoV or rotaviruses, unless there is an inactivating mutation of their 2-5A-degrading enzymes (NS2, NS4b, and VP3, respectively) (22, 24–26). In contrast, SARS-CoV-2, which lacks a gene for a similar protein, is inhibited by RNase L (4). In this context, enzymes that degrade 2-5A, such as PDE12, are drug targets in the hunt for broad-spectrum antiviral agents (32, 47).

The viral enzymes NS2, NS4b, and VP3-CTD are antagonists of innate immunity that support virus replication by eliminating 2-5A and preventing, or reducing, the activation of RNase L by 2-5A (20, 22, 24–26). In contrast, mammalian AKAP7 is a nuclear 2′,5′-PE that does not affect viral replication unless its nuclear localization signal peptide is deleted, leading to cytoplasmic accumulation (27). A mutant AKAP7 deleted for its N-terminal nuclear localization signal peptide accumulates in the cytoplasm and was able to rescue an NS2 mutant of MHV (22). While the function of the 2′,5′-oligonucleotide-cleaving activity of AKAP7 is still unresolved, the phylogenetic tree suggests that the NS2 coronavirus proteins may have evolved from the AKAP7 catalytic domain (Fig. 2A).

Enzymes that degrade 2-5A have significance beyond antiviral innate immunity. Self-dsRNA activates the OAS-RNase L pathway, leading in some circumstances to apoptosis (12, 13). In one example, mutation or inhibition of the dsRNA editing enzyme ADAR1 leads to the accumulation of self-dsRNA activating OAS-RNase L, leading to cell death, and protein kinase R (PKR), inhibiting protein synthesis initiation (16, 48). In another instance, DNA methyltransferase inhibitors, e.g., 5-aza-cytidine, cause self-dsRNA accumulation from repetitive DNA elements, leading to OAS-RNase L activation and apoptosis (17, 49). Thus, 2-5A is a secondary messenger for cytotoxic and antiviral activities of either nonself dsRNA (viral) or self-dsRNA (host), whose levels must be tightly controlled to limit cytotoxicity while restricting viral spread. Our findings provide a mechanistic understanding of how 2′,5′-PEs regulate 2-5A levels among the coronaviruses MHV and MERS-CoV, group A rotaviruses, and mammalian cells through the activity of AKAP7 (22, 24, 25, 27), with implications for both the control of virus replication and cellular responses to self-dsRNA. Furthermore, our study defines 2′,5′-PEs as a new subgroup within the 2H-PE superfamily that shares characteristic conserved sequence features of the superfamily but with specific and distinct biochemical cleavage activities. Knowledge derived from the study of these 2-5A-degrading enzymes could lead to future avenues of antiviral drug development.

## MATERIALS AND METHODS

### cDNA cloning and plasmids.

Human PDE12 cDNA (GenBank accession number NM_177966.5) was PCR amplified (using DNASU cDNA clone HsCD00296464 in vector pDONR221) with forward primer 5′-TTCAAgaattcATGTGGAGGCTCCCAGGCGC-3′ (with an EcoRI restriction site [in underlined lowercase type]) and reverse primer 5′-TTCAAgtcgacTCATTTCCATTTTAAATCACATACAAGTGCTATGTGATC-3′ (with a SalI restriction site [in underlined lowercase type]). The PDE12^E351A^ pGEX 6P-1 mutant (34) plasmid was constructed by the MegaPrimer method (50) using mutagenic reverse primer 5′-GCGCGGTCAACCgCCTGCAAACAG-3′, where “g” represents mutated nucelotide. The amplified wild-type and mutant PDE12 cDNAs were cloned into plasmid pGEX-6P-1 (GE Healthcare, USA) at the EcoRI and SalI restriction site, sequenced, and expressed in Escherichia coli as glutathione *S*-transferase (GST) fusion proteins. To subclone the VP3 C-terminal domain (CTD) cDNA of rotavirus A strain RVA/Simian-tc/USA/RRV/1975/G3P (GenBank accession number EU636926.1) and its H718A mutant, we used codon-optimized constructs for expression in Sf9 insect cells (GenBank accession number KJ869109.1) (30) (cDNA tempelates were gifts from Kristin Ogden, Vanderbilt University). The cDNAs were PCR amplified and cloned into plasmid pMAL-C5X at the XmnI (blunt cloned) and NcoI (sticky end) restriction sites. Blunt-end forward primer 5′-TACGCTGACGACCCCAACTACTTCATCG-3′ and reverse primer 5′-TTCAAccatggTTATTACTCGGACATGTCGAACACGGTGTCG-3′ with an NcoI restriction site (in underlined lowercase type) were used for VP3-CTD. The wild-type and H718A RVA VP3-CTD proteins were expressed as fusions to maltose binding protein (MBP). Additional protein expression plasmid constructs were previously described, with sequences originating from MERS-CoV (MBP-NS4b and its mutant MBP-NS4b^H182R^) (24), MHV (MBP-MHV NS2 and its mutant MBP-NS2^H126R^) (22), and mouse AKAP7 and its mutant AKAP7^H93A;H185R^ (27).

### Protein expression and purification.

Proteins were expressed from pGEX-6P-1 or pMAL constructs in E. coli strain BL21(DE3)/pLysS (Life Technologies, USA). Wild-type and catalytically inactive mutants of AKAP7 and PDE12 were expressed as GST fusion proteins, and purification was performed by modification of a previous protocol (51). Single colonies were used to inoculate primary cultures, which were subsequently used to seed secondary cultures grown to an optical density (OD) (at 600 nm) of 0.6 in a shaking incubator at 37°C at 250 rpm. Cells were induced with 0.2 mM isopropyl-β-d-thiogalactopyranoside (IPTG) for 16 h at 22°C. Induced cell pellets were resuspended in buffer A (20 mM HEPES [pH 7.5], 1 M KCl, 1 mM EDTA, 10% [vol/vol] glycerol, 5 mM dithiothreitol [DTT], and EDTA-free Pierce protease inhibitor [Thermo Scientific, USA]). Pelleted cells were lysed by the addition of 200 μg/ml lysozyme, followed by sonication. Supernatants were collected after centrifugation at 12,000 × *g* for 40 min at 4°C in a Beckman JA-17 rotor. Supernatants were added to Pierce glutathione agarose (Thermo Scientific, USA) and incubated for 2 h at 4°C, followed by washes with buffer A. Digestions to release the GST tag were performed with PreScission protease (Cytiva, USA) in a solution containing 50 mM Tris-HCl (pH 7.5), 150 mM NaCl, 1 mM EDTA, and 1 mM DTT for 16 h at 4°C. Supernatants containing untagged protein were concentrated using Centriprep centrifugal filter devices (molecular weight cutoff of 10 kDa; Millipore) and loaded onto a Superdex 75 column on an Äkta pure 25L protein purification system (GE Healthcare, USA) in a solution containing 20 mM HEPES (pH 7.5), 150 mM NaCl, and 1 mM DTT. Wild-type and mutant RVA VP3-CTD-expressing bacterial culture growth and IPTG induction conditions were the same as the ones described above, except that growth medium additionally included 2% glucose. Harvested bacterial cell pellets were suspended in buffer B (20 mM Tris-HCl [pH 7.4] with 200 mM NaCl, 1 mM EDTA, 10 mM β-mercaptoethanol, an EDTA-free protease inhibitor [Pierce protease inhibitor; Thermo Scientific, USA], and 10% glycerol) and lysed with lysozyme, followed by sonication. Supernatants were incubated with amylose resin (New England BioLabs [NEB], USA) and washed three times with buffer, followed by elution with 100 mM maltose. Proteins were concentrated using Centriprep centrifugal filter devices (molecular weight cutoff of 10 kDa; Millipore) and further purified using size exclusion chromatography (SEC) on an Äkta pure 25L protein purification system (GE Healthcare, USA) in buffer C (20 mM HEPES [pH 7.5], 100 mM NaCl, and 1 mM DTT). Wild-type and catalytic mutants of NS4b and MHV NS2 were purified as described previously (22, 24). In addition to inactive mutants, purified MBP was used as the control in experiments with MBP fusion proteins. Protein concentrations were determined using the Bio-Rad protein assay reagent (Bio-Rad, USA). All proteins were stored in buffer C supplemented with 10% glycerol at −80°C.

### Synthesis and purification of 2-5A oligomers and other oligoribonucleotide substrates.

2-5A or p_3_5′A(2′p5′A)_2_ (2′,5′-p_3_A_3_) was synthesized from ATP by using histidine-tagged porcine OAS1 (52). The OAS was immobilized and activated with poly(I):poly(C)-agarose (53). Briefly, poly(I):poly(C)-agarose beads were washed with buffer D [10 mM HEPES (pH 7.5), 1.5 mM Mg(CH_3_COO)_2_·4H_2_O, 50 mM KCl, 20% glycerol, and 7 mM β-mercaptoethanol]. Ten milliliters of beads was incubated with 10 mg of purified OAS protein for 2 h at 25°C, with intermittent vortexing. Beads were washed three times with buffer D by centrifugation at 3,000 × *g* at 4°C for 30 min. Beads were suspended in reaction mixtures containing 20 mM HEPES (pH 7.5), 20 mM Mg(CH_3_COO)_2_·4H_2_O, 20 mM KCl, 1 mM EDTA, and 10 mM ATP. The reaction mixtures were incubated in a shaking incubator set at 37°C at 120 rpm for 18 h. The supernatant was collected by centrifugation at 3,000 × *g* at 4°C for 30 min. The supernatant was heated at 95°C for 5 min and again centrifuged at 18,000 × *g* for 15 min at 4°C to remove the precipitate. To isolate individual 2-5A oligomers, the supernatants containing crude, unfractionated 2-5A oligomers were run on an HPLC instrument (1260 Infinity II; Agilent Technologies) equipped with a preparative Dionex column (BioLC DNAPac PA-100, 22 by 250 mm; Dionex, USA). Samples were injected and elution was performed at a flow rate of 3 ml/min in a stepwise gradient of 10 to 400 mM (0 to 120 min), 400 to 800 mM (121 to 125 min), and 10 mM (126 to 160 min) NH_4_HCO_3_ buffer (pH 7.8). Fractions were collected, lyophilized, and suspended in nuclease-free water.

RNA oligoribonucleotides (other than 2′,5′-p_3_A_3_) with 2′-5′- or 3′-5′-phosphodiester linkages were commercially purchased. The oligonucleotide substrates 5′-pA2′p5′A2′p5′A2′p5′A2′p5′A-3′, 5′-pU2′p5′U2′p5′U2′p5′U2′p5′U-3′, 5′-pG2′p5′G2′p5′G2′p5′G2′p5′G-3′, 5′-pA3′p5′A3′p5′A3′p5′A3′p5′A-3′, and 5′-pU3′p5′U3′p5′U3′p5′U3′p5′U-3′ were purchased from Integrated DNA Technologies (IDT), while 5′-pC2′p5′C2′p5′C2′p5′C2′p5′C-3′, 5′-pC3′p5′C3′p5′C3′p5′C3′p5′C-3′, and 5′-pA2′p5′A-3′ were purchased from ChemGenes Corporation (Wilmington, MA, USA). Pentaribonucleotide substrates are shown as p5′(rN)_5_, where N represents an A, U, G, or C nucleotide. The A2′p5′A standard was prepared by incubating 5′pA2′p5′A with shrimp alkaline phosphatase (SAP; Thermo Fisher, USA) according to the manufacturer’s protocol. 2′,3′-Cyclic GMP-AMP (cGAMP), ATP, AMP, and adenosine were obtained from Sigma-Aldrich.

### Phosphodiesterase activity assays.

A total of 10 μM the substrates (with either a 2′-5′- or 3′-5′-phosphodiester linkage) was incubated with 1 μM enzyme. Final reactions were performed in a solution containing 20 mM HEPES buffer (pH 7.4), 1 mM DTT, and 10 mM MgCl_2_ by incubation at 30°C for 1 h (or for the time indicated in the text). Where indicated, reactions were performed in the absence of MgCl_2_ with 2 mM EDTA added. Reactions were stopped by heating at 95°C for 5 min. Samples were centrifuged at 18,000 × *g* for 15 min at 4°C. Supernatants were collected and analyzed by HPLC. 2′,3′-cGAMP degradation assays were performed and analyzed under the same conditions as the ones described above. In all experiments, substrates incubated under similar conditions in the absence of enzyme served as controls.

### HPLC analysis and identification of products.

The substrates and cleavage products were analyzed on a 1260 Infinity II Agilent Technologies HPLC instrument equipped with an Infinitylab Poroshell 120 C_18_ analytical column (4.6 by 150 mm, 4 μm; Agilent Technologies). Eluent A was 50 mM ammonium phosphate buffer (pH 6.8), and eluent B was 50% methanol in water. Five microliters of processed samples was injected onto the C_18_ column, at a flow rate of 1 ml/min, and eluted with a linear gradient (0 to 40%) of eluent B over a period of 20 min and then 3 min of 40% eluent B, followed by equilibration to initial conditions (100% eluent A). The HPLC column was maintained at 40°C. Spectra were recorded at 256 nm. The products were identified either by comparison to the elution times of known standards or by mass spectrometry analysis. Alternatively, to test expanded substrate specificity, 10 μl of the processed samples was injected onto a Dionex DNAPac PA-100 analytical column at a flow rate of 1 ml/min and eluted with a linear gradient of 10 to 800 mM NH_4_HCO_3_ buffer (pH 7.8) over a period of 90 min, followed by 30 min of equilibration to initial conditions. Open Lab CDS software was used to analyze and calculate the areas under the peaks in HPLC spectra.

### Shrimp alkaline phosphatase-mediated phosphorylation analysis.

Purified substrates and cleavage product mixtures were dephosphorylated by incubation with SAP (Thermo Fisher, USA) at 37°C for 1 h according to the manufacturer’s protocol. Samples were prepared for subsequent analysis as described above.

### Sample preparation for mass spectrometry.

The desired peak fractions (including cleavage products of 2′,5′-p_3_A_3_) were collected by running samples on a Dionex DNAPac PA-100 analytical column as described above. The collected peaks were subjected to acetone precipitation, and supernatants containing cleavage products (from the HPLC peak) were collected and lyophilized. Lyophilized samples were suspended in 1 mM NH_4_HCO_3_ buffer (pH 7.8) and used for mass spectrometry analysis.

### Mass spectrometry analysis of cleavage products.

The prepared samples were subjected to mass spectrometry analysis. The LC-MS/MS analysis was carried out using a triple-quadrupole tandem mass spectrometer (TSQ-Quantiva; Thermo Scientific, USA) equipped with an electrospray ionization (ESI) interface. The mass spectrometer was coupled to the outlet of the HPLC system that consisted of an ultrahigh-performance liquid chromatography (UHPLC) system (Vanquish; Thermos Fisher Scientific, USA) including an autosampler with a refrigerated sample compartment and an inline vacuum degasser. Xcalibur software was used for data processing. ESI mass spectrometric detection was performed in both the negative and positive ionization modes, with an ion spray voltage at 2.5 kV, sheath gas at 35 Arb (arbitrary relative unit for measurement of gas flow), and auxiliary (Aux) gas at 20 Arb. The ion transfer tube and vaporizer temperatures were set at 350°C and 250°C, respectively. Qualitative analysis was performed using a full scan at a range from *m/z* 200 to 1,250. Five-microliter extracted samples were injected onto the C_18_ column (Gemini, 3 μm, 2 by 150 mm; Phenomenex, CA) with a flow rate of 0.3 ml/min at 45°C. Mobile phases were mobile phase A (water containing 10 mM ammonium acetate and 20 mM ammonium hydroxide) and mobile phase B (methanol containing 10 mM ammonium acetate and 20 mM ammonium hydroxide). Mobile phase B at 0% was used at 0 to 2 min, and linear gradients were used starting from 0% mobile phase B to 100% B at 2 to 12 min and kept at 100% at 12 to 26 min and then from 100% B to 0% B at 26 to 27 min and kept at 0% mobile phase B for 8 min. The peaks shown in full scans were processed to locate and identify the cleavage products of the 2′,5′-p_3_A_3_ substrate using Xcalibur software v4.1. The standards adenosine, AMP, ATP, and adenosine-2′,3′-cyclic monophosphate sodium salt were run for reference.

### Bioinformatic analysis.

The PDE domain sequences from different 2′,5′-PEs were used for creating a multiple-sequence alignment using MAFFT (Multiple Alignment using Fast Fourier Transform) version 7 (54), employing the E-INS-I iterative refinement method (https://mafft.cbrc.jp/alignment/server/). The MAFFT sequence alignment result was downloaded in Clustal format and visualized using Jalview 2.11.1.3 software. The sequence alignment was further processed on the MAFFT server to calculate the phylogenetic tree using the neighbor-joining method and the JTT (Jones-Taylor-Thornton) substitution model, and the tree was then visualized using Archaeopteryx.js software. The resultant fasta format output of the MAFFT multiple-sequence alignment was used to calculate the percentages of amino acid identity and similarity by the Sequence Identity and Similarity (SIAS) tool with default parameters (http://imed.med.ucm.es/Tools/sias.html). The names (accession numbers, amino acid regions) of the aligned sequences are MHV NS2 (UniProtKB/Swiss-Prot accession number P19738.1, amino acids [aa] 41 to 135), human coronavirus (HCoV) OC43 NS2 (GenBank accession number AAT84352.1, aa 43 to 138), human enteric coronavirus (HECoV) NS2 (GenBank accession number ACJ35484.1, aa 39 to 140), equine coronavirus (ECoV) NS2 (GenBank accession number ABP87988.1, aa 42 to 140), Middle East respiratory syndrome coronavirus (MERS-CoV) NS4b (GenBank accession number AFS88939.1, aa 87 to 191), rat AKAP7δ/γ (NCBI RefSeq accession number NP_001001801.1, aa 121 to 233), mouse AKAP7 isoform 1 (NCBI RefSeq accession number NP_061217.3, aa 82 to 194), human AKAP7γ (NCBI RefSeq accession number NP_057461.2, aa 100 to 233), human rotavirus group A (RVA) WA-VP3 (GenBank accession number AFR77808.1, aa 707 to 806), simian RVA SA11-N5 (GenBank accession number AFK09591.1, aa 707 to 808), human rotavirus group B (RVB) Bang117 (GenBank accession number ADF57896.1, aa 655 to 750), bat coronavirus (BtCoV) SC2013 NS4b (GenBank accession number AHY61340.1, aa 96 to 195), and BtCoV HKU5 NS4b (NCBI RefSeq accession number YP_001039965.1, aa 91 to 192).

### Data availability.

Multiple-sequence alignment software is available at https://mafft.cbrc.jp/alignment/server/. The alignment and phylogenetic tree construction tool is downloadable from https://www.jalview.org/. The Sequence Identity and Similarity (SIAS) tool with default parameters is available at http://imed.med.ucm.es/Tools/sias.html. The names (accession numbers) of the aligned sequences are MHV NS2 (UniProtKB/Swiss-Prot accession number P19738.1), HCoV OC43 NS2 (GenBank accession number AAT84352.1), HECoV NS2 (GenBank accession number ACJ35484.1), ECoV NS2 (GenBank accession number ABP87988.1), MERS-CoV NS4b (GenBank accession number AFS88939.1), rat AKAP7δ/γ (NCBI RefSeq accession number NP_001001801.1), mouse AKAP7 isoform 1 (NCBI RefSeq accession number NP_061217.3), human AKAP7γ (NCBI RefSeq accession number NP_057461.2), human RVA WA VP3 (GenBank accession number AFR77808.1), simian RVA SA11 N5 (GenBank accession number AFK09591.1), human RVB Bang117 (GenBank accession number ADF57896.1), BtCoV SC2013 NS4b (GenBank accession number AHY61340.1), and BtCoV HKU5 NS4b (NCBI RefSeq accession number YP_001039965.1).
