# Supplementary material for: Specificity and Mechanism of Coronavirus, Rotavirus, and Mammalian Two-Histidine Phosphoesterases That Antagonize Antiviral Innate Immunity
Source: mBio. 2021 Aug 10;12(4):e01781-21. doi: 10.1128/mBio.01781-21 (PMC8406329; doi:10.1128/mBio.01781-21)
Supplement: TABLE S1 [file mbio.01781-21-st001.pdf]

amino acid identity (%)

Group		MHV NS2	HCoV OC43 NS2	HECoV NS2	ECoV NS2	Rat AKAP7 δ/γ	Mu AKAP7 isoform-1	Hu AKAP7 γ	BtCoV HKU5 NS4b	BtCoV SC2013 NS4b	MERS-CoV NS4b	Hu RVA WA VP3	Simian RVA SA11	Hu RVB Bang117
NS2	MHV NS2		57.9	56.8	48.4	17.9	18.9	16.8	14.7	16.8	11.6	20.0	22.1	21.1
	HCoV OC43 NS2	74.7		92.6	61.1	21.1	22.1	18.9	11.6	16.8	14.7	22.1	20.0	21.1
	HECoV NS2	72.6	94.7		60.6	18.6	19.6	17.6	11.8	16.0	13.7	20.0	18.8	20.8
	ECoV NS2	64.2	71.6	72.7		19.2	19.2	19.2	13.1	16.2	12.1	16.2	18.2	17.7
AKAP7	Rat AKAP7 δ/γ	31.6	35.8	33.3	35.4		97.3	85.8	12.7	16.0	18.1	19.0	18.8	11.5
	Mu AKAP7 isoform-1	32.6	36.8	34.3	35.4	97.3		85.0	12.7	17.0	18.1	19.0	18.8	12.5
	Hu AKAP7 γ	30.5	33.7	33.3	36.4	89.4	88.5		12.7	17.0	18.1	20.0	19.8	13.5
NS4b	BtCoV HKU5 NS4b	29.5	27.4	27.5	29.3	21.6	21.6	22.5		41.0	35.3	11.0	10.9	12.5
	BtCoV SC2013 NS4b	27.4	29.5	27.0	26.3	27.0	28.0	29.0	52.0		49.0	19.0	13.0	15.6
	MERS-CoV NS4b	24.2	27.4	25.5	28.3	26.7	26.7	27.6	50.0	69.0		18.0	15.8	10.4
VP3	Hu RVA WA VP3	30.5	29.5	27.0	26.3	24.0	24.0	25.0	26.0	30.0	34.0		78.0	16.7
	Simian RVA SA11 VP3	32.6	30.5	28.7	28.3	23.8	23.8	24.8	23.8	26.0	31.7	84.0		16.7
	Hu RVB Bang117	30.5	32.6	32.3	27.1	19.8	20.8	25.0	29.2	27.1	24.0	29.2	29.2	

amino acid similarity (%)

Table S1. Catalytic domain sequence identity and similarity analysis of viral and cellular 2’,5’-PEs. Percent amino acid identity and similarity matrix is based on alignment in figure 2B. 2’,5’-PEs from mammals or mammalian viruses were used for alignment. Values are calculated using Sequence Identity and Similarity (SIAS) tool. Matrix values show percent identity (above diagonal) and similarity (below diagonal) between the corresponding pair of the proteins. Intragroup identity and similarity values are shaded in grey.
