# Supplementary material for: Specificity and Mechanism of Coronavirus, Rotavirus, and Mammalian Two-Histidine Phosphoesterases That Antagonize Antiviral Innate Immunity
Source: mBio. 2021 Aug 10;12(4):e01781-21. doi: 10.1128/mBio.01781-21 (PMC8406329; doi:10.1128/mBio.01781-21)
Supplement: FIG S1 [file mbio.01781-21-sf001.pdf]

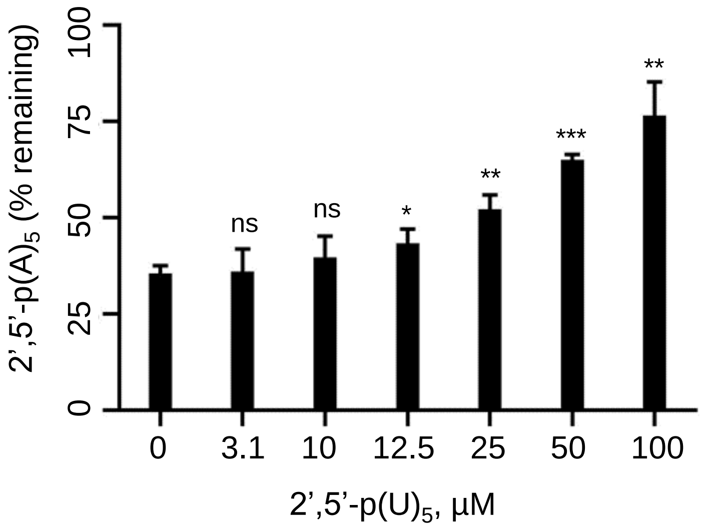

Figure S1. 2’,5’-p(A)$_5$ degradation by MHV NS2 decreases in the presence of 2’,5’-p(U)$_5$. Substrate 2’,5’-p(A)$_5$ was incubated with 0.2 µM of MHV-NS2 wild type protein in the absence or presence of indicated concentration of 2’,5’-p(U)$_5$ at 30°C for 10 min. Samples were processed and analyzed by HPLC. 2’,5’-p(A)$_5$ incubated under similar conditions in the absence of MHV-NS2 and 2’,5’-p(U)$_5$ served as non-degraded control. Experiments were performed three times (n=3) and bars represent the standard error of mean. Statistical significance was calculated using unpaired t test (n=3; *, P value < 0.05; **, P < 0.005;***, P < 0.001; ns, not significant) in GraphPad Prism (9.0.0) software.
