# Supplementary material for: Specificity and Mechanism of Coronavirus, Rotavirus, and Mammalian Two-Histidine Phosphoesterases That Antagonize Antiviral Innate Immunity
Source: mBio. 2021 Aug 10;12(4):e01781-21. doi: 10.1128/mBio.01781-21 (PMC8406329; doi:10.1128/mBio.01781-21)
Supplement: TABLE S2 [file mbio.01781-21-st002.pdf]

Substrate	2',5'-PE activity (% substrate degraded)							
	MERS NS4b		MHV NS2		RVA VP3-CTD		muAKAP7	
	WT	H182R	WT	H126R	WT	H718A	WT	H93A;H185R
2'-5' oligonucleotides								
p5'$(rA)_5$	>99	<1	>99	<1	>95	3	>99	<1
p5'$(rU)_5$	4	1.2	<1	<1	40	<1	>95	<1
p5'$(rC)_5$	<1	<1	7	<1	90	<1	>95	<1
p5'$(rG)_5$	<1	<1	<1	<1	6	<1	>90	~40
3'-5' oligonucleotides								
p5'$(rA)_5$	<1	<1	<1	<1	<1	<1	<1	<1
p5'$(rU)_5$	<1	<1	<1	<1	<1	<1	<1	<1
p5'$(rC)_5$	<1	<1	<1	<1	<1	<1	<1	<1

Table S2. MERS-NS4b, MHV NS2, RVA VP3-CTD and muAKAP7 mediated degradation of 5'-phosphorylated, 2'-5' or 3'-5' linked penta-ribonucleotide substrates. Ten µM of the indicated substrate was incubated with 1 µM of wild type or mutant 2',5'-PE for 1 h at 30°C. Percent substrate degradation was calculated by measuring the area under the peaks in the HPLC chromatograms. Results were reproduced in at least two independent experiments.
