# Supplementary material for: Specificity and Mechanism of Coronavirus, Rotavirus, and Mammalian Two-Histidine Phosphoesterases That Antagonize Antiviral Innate Immunity
Source: mBio. 2021 Aug 10;12(4):e01781-21. doi: 10.1128/mBio.01781-21 (PMC8406329; doi:10.1128/mBio.01781-21)
Supplement: TABLE S3 [file mbio.01781-21-st003.pdf]

**MERS-NS4b (3 µM) mediated degradation of 2'-5' linked penta-ribonucleotides substrates (10 µM) (% substrate degraded).**		
Substrate	NS4b WT	$NS4b^{H182R}$
$p5'(rA)_5$	>99	<1
$p5'(rU)_5$	5	<1
$p5'(rC)_5$	6	2
$p5'(rG)_5$	<1	<1

Table S3. MERS-NS4b mediated degradation of 5'-phosphorylated 2'-5' or 3'-5' linked penta-ribonucleotide substrates. Ten µM of the indicated substrate was incubated with 3 µM of wild type or mutant MERS-NS4b for 1 h at 30°C. Percent substrate degradation was calculated by measuring the area under the peaks in the HPLC chromatograms. Results were reproduced in two independent experiments.
