# Supplementary material for: Specificity and Mechanism of Coronavirus, Rotavirus, and Mammalian Two-Histidine Phosphoesterases That Antagonize Antiviral Innate Immunity
Source: mBio. 2021 Aug 10;12(4):e01781-21. doi: 10.1128/mBio.01781-21 (PMC8406329; doi:10.1128/mBio.01781-21)
Supplement: TABLE S4 [file mbio.01781-21-st004.pdf]

2’,5’-PE activity (% degradation of the indicated substrate)								
Substrate	MERS NS4b		MHV NS2		RVA VP3-CTD		muAKAP7	
	WT	H182R	WT	H126R	WT	H718A	WT	H93A;H185R
2’,3’-cGAMP	<1	<1	<1	<1	<1	<3	<3	<5
2’,5’-p_3A_3	>99	<2	>98	<5	>98	<2	>99	<1

Table S4. 2’,5’-PEs mediated degradation of 2’,3’-cGAMP and 2’,5’-p_3A_3. Ten µM of the indicated substrate was incubated with 1 µM of wild type or mutant 2’,5’-PEs for 1 h at 30°C. Substrate without enzyme incubated under similar condition were used as un-degraded control. Percent substrate degradation was calculated by measuring the area under the peaks in the HPLC chromatograms. Results were reproduced in two independent experiments.
