# Supplementary material for: Specificity and Mechanism of Coronavirus, Rotavirus, and Mammalian Two-Histidine Phosphoesterases That Antagonize Antiviral Innate Immunity
Source: mBio. 2021 Aug 10;12(4):e01781-21. doi: 10.1128/mBio.01781-21 (PMC8406329; doi:10.1128/mBio.01781-21)
Supplement: FIG S2 [file mbio.01781-21-sf002.pdf]

Substrate + MERS-NS4b

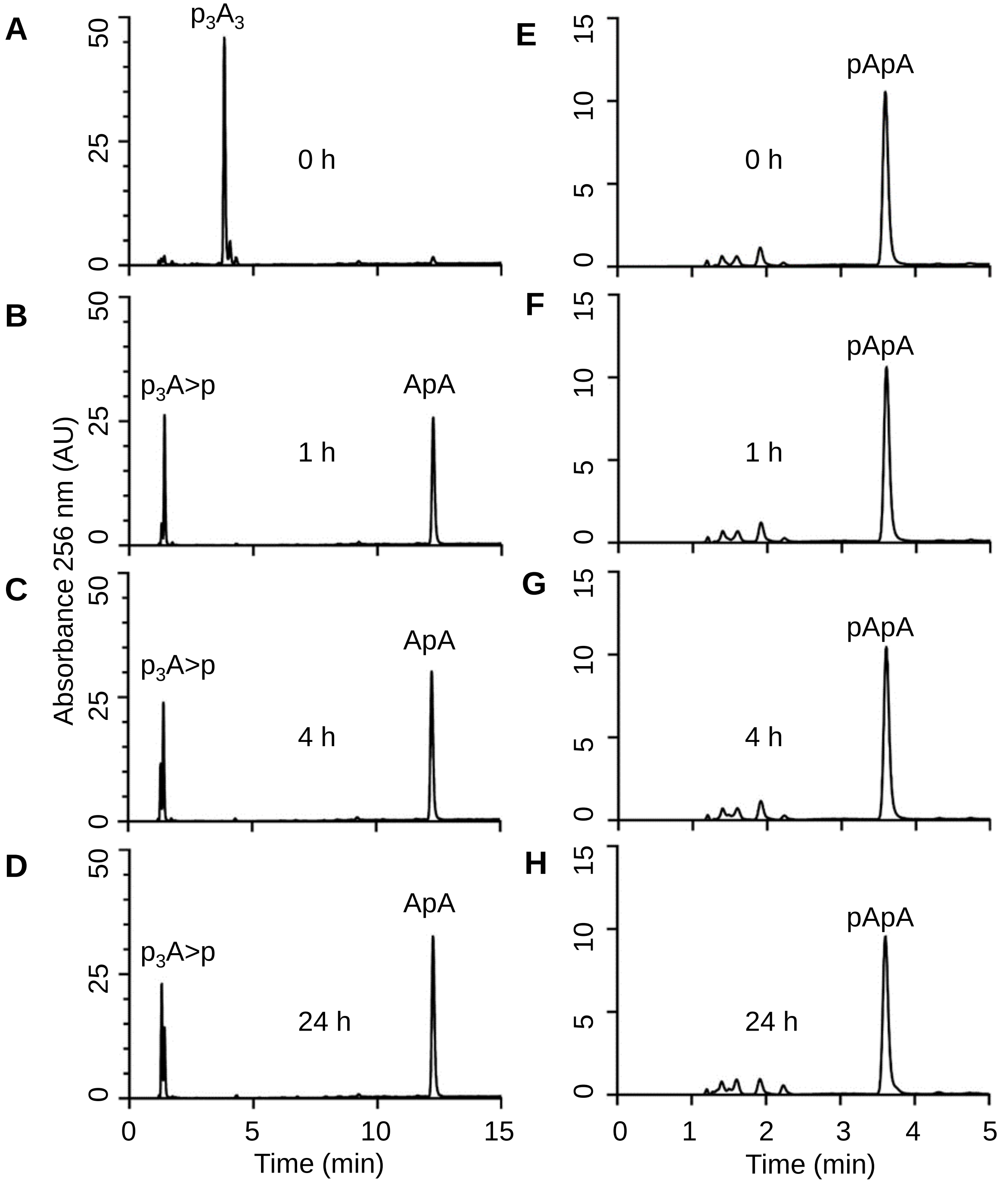

I

Time (h)	p_3A_3	ApA (cleavage product)	pApA
0	100	N/A	100
1	<1	100	>99
4	<1	>98	>99
24	<1	>98	>99

* ApA was formed upon p_3A_3 degradation by NS4b. Area under the peak at 10 min was used as reference (100 %) to compare its degradation with time.

Figure S2. MERS-NS4b degrades 2',5'-p_3A_3 but not 2',5' linked ApA or pApA. Substrate 2',5'-p_3A_3 (A) was degraded in the presence of NS4b into p_3A>p and ApA (B). Panels B, C, and D showed no appreciable decrease in amounts of ApA (area under the peak) after incubation for 1, 4 and 24 h, respectively. Panels E, F, G and H shows HPLC chromatograms of substrate pApA in the presence for NS4b at 0, 1, 4 and 24 h. (I) The table shows the amount of ApA or pApA degraded by NS4b as a function of time.
