# Supplementary material for: Specificity and Mechanism of Coronavirus, Rotavirus, and Mammalian Two-Histidine Phosphoesterases That Antagonize Antiviral Innate Immunity
Source: mBio. 2021 Aug 10;12(4):e01781-21. doi: 10.1128/mBio.01781-21 (PMC8406329; doi:10.1128/mBio.01781-21)
Supplement: FIG S3 [file mbio.01781-21-sf003.pdf]

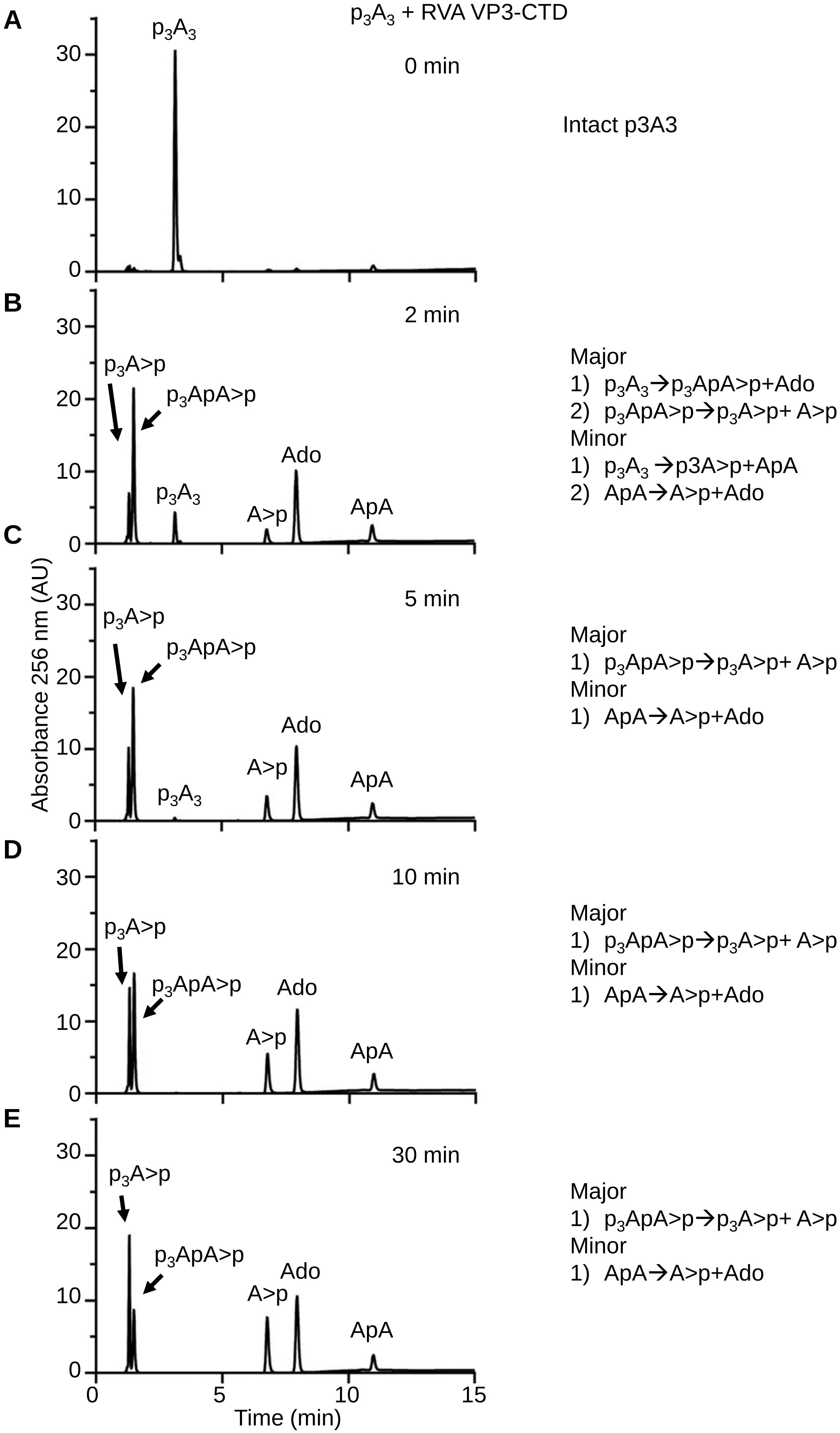
A
p_3A_3 + RVA VP3-CTD
p_3A_3
0 min
Intact p3A3
B
2 min
$p_3A{>}p$
$p_3ApA{>}p$
p_3A_3
A>p
Ado
ApA
Major
1) $p_3A_3 \rightarrow p_3ApA{>}p$+Ado
2) $p_3ApA{>}p \rightarrow p_3A{>}p$+ A>p
Minor
1) $p_3A_3 \rightarrow$p3A>p+ApA
2) ApA$\rightarrow$A>p+Ado
C
5 min
$p_3A{>}p$
$p_3ApA{>}p$
p_3A_3
A>p
Ado
ApA
Major
1) $p_3ApA{>}p \rightarrow p_3A{>}p$+ A>p
Minor
1) ApA$\rightarrow$A>p+Ado
D
10 min
$p_3A{>}p$
$p_3ApA{>}p$
A>p
Ado
ApA
Major
1) $p_3ApA{>}p \rightarrow p_3A{>}p$+ A>p
Minor
1) ApA$\rightarrow$A>p+Ado
E
30 min
$p_3A{>}p$
$p_3ApA{>}p$
A>p
Ado
ApA
Major
1) $p_3ApA{>}p \rightarrow p_3A{>}p$+ A>p
Minor
1) ApA$\rightarrow$A>p+Ado
Absorbance 256 nm (AU)
0
10
20
30
0
5
10
15
Time (min)

Figure S3. Time-course of 2',5'-p_3A_3 cleavage by RVA VP3-CTD. Purified 2',5'-p_3A_3 (200 μM) was incubated with RVA VP3-CTD (0.05 μM) at 30°C. Samples were collected at (A) 0 min, (B) 2 min, (C) 5 min, (D) 10 min, and (E) 30 min and analyzed by HPLC. The percent of substrate or products at indicated times were determined by calculating the area under the peaks on the HPLC chromatograms. Right hand side shows major and minor reactions proceeding at the indicated time points deduced from HPLC chromatogram analysis.
