# Supplementary material for: Specificity and Mechanism of Coronavirus, Rotavirus, and Mammalian Two-Histidine Phosphoesterases That Antagonize Antiviral Innate Immunity
Source: mBio. 2021 Aug 10;12(4):e01781-21. doi: 10.1128/mBio.01781-21 (PMC8406329; doi:10.1128/mBio.01781-21)
Supplement: FIG S4 [file mbio.01781-21-sf004.pdf]

**A** Percentage population of different species from experiment in figure S2 involving RVA VP3-CTD is calculated by integrating the area under the peak on chromatogram during the reaction.

| **Time (min)** | **$p_3A_3$** | **$p_3A{>}p$** | **$p_3ApA{>}p$** | **A>p** | **Ado** | **ApA** |
|---|---|---|---|---|---|---|
| 0 | 100 | - | - | - | - | - |
| 1 | 24 | 8 | 33 | 4 | 24 | 6.3 |
| 2 | 9 | 11 | 38 | 6 | 28 | 6.7 |
| 5 | - | 15 | 35 | 10.5 | 30.7 | 6.5 |
| 10 | - | 18.5 | 28.5 | 15 | 31 | 6.5 |
| 20 | - | 24 | 19.2 | 19.6 | 30.8 | 6.5 |
| 30 | - | 25.8 | 16.5 | 21.5 | 30.4 | 6.5 |
| 45 | - | 25.9 | 14.7 | 22.5 | 30.3 | 6.4 |
| 60 | - | 26.8 | 13.8 | 22.9 | 30.3 | 6.4 |

**B**

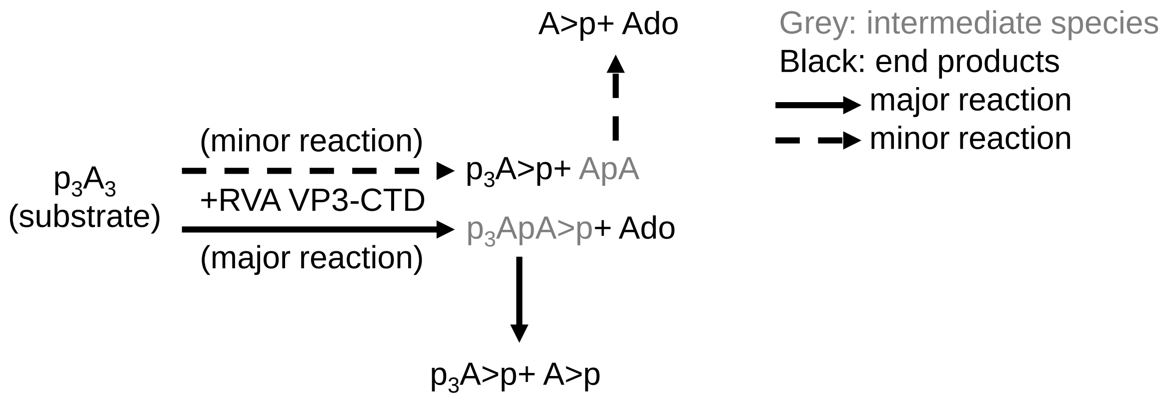


**Figure S4. Mechanism of 2',5'-$p_3A_3$ cleavage by RVA VP3-CTD.** (A) The percentage of the substrate or the products at indicated times were determined by calculating the area under the peaks on the HPLC chromatograms obtained in experiment from figure S3 . (B) Summary of major and minor reactions involved in cleavage of 2',5'-$p_3A_3$ by VP3-CTD. The minor reaction cleavage of ApA to A>p and Ado is inferred from incubations performed at a 20-fold higher concentration of RVA VP3-CTD, 1 μM (Fig. 3D).
