# Supplementary material for: Specificity and Mechanism of Coronavirus, Rotavirus, and Mammalian Two-Histidine Phosphoesterases That Antagonize Antiviral Innate Immunity
Source: mBio. 2021 Aug 10;12(4):e01781-21. doi: 10.1128/mBio.01781-21 (PMC8406329; doi:10.1128/mBio.01781-21)
Supplement: FIG S5 [file mbio.01781-21-sf005.pdf]

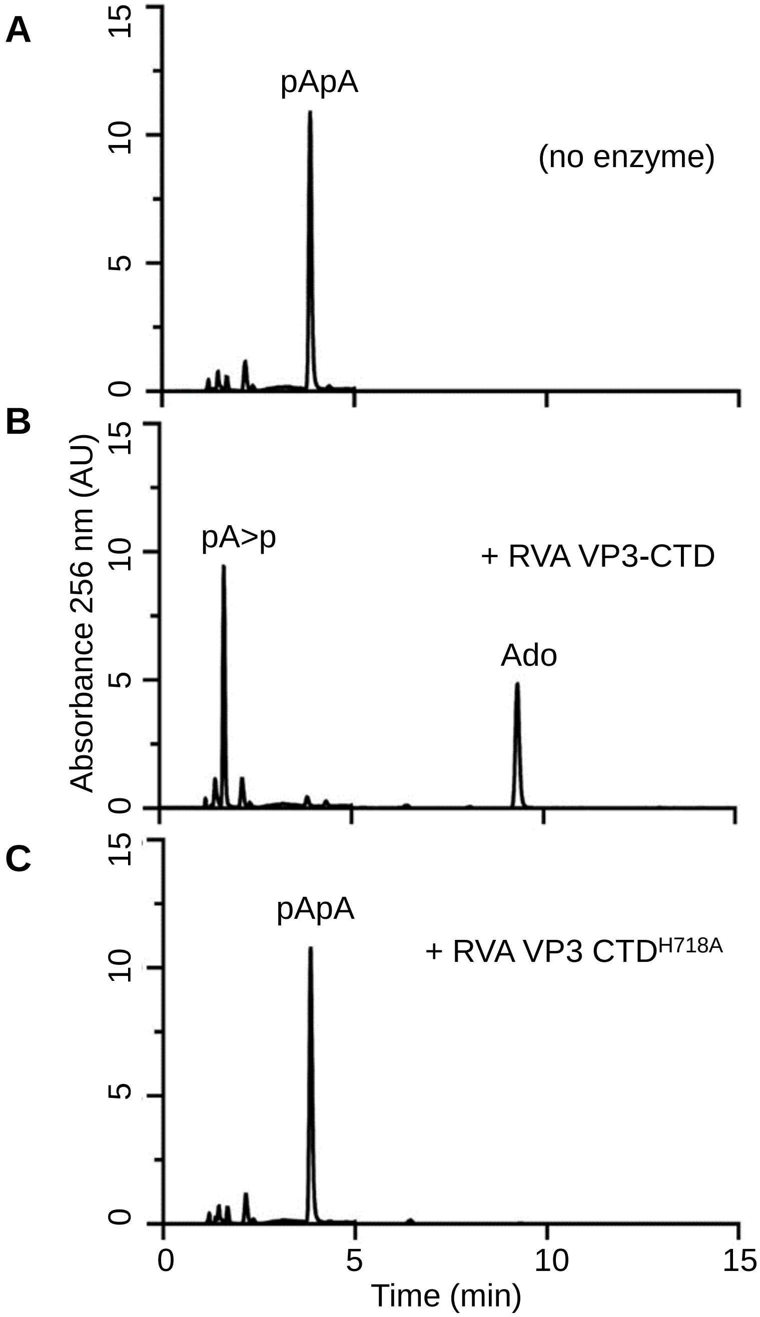

Figure S5. RVA VP3-CTD degrades 2',5' linked di-adenylate. (A) Substrate 2',5'-pApA (A) was incubated with 1 μM of either (B) wild type RVA VP3-CTD or its mutant (C) RVA VP3 CTDH^{718A} at 30°C for 1 h. Samples were processed and analyzed by HPLC. Baseline buffer signal was subtracted from the samples.
