# Supplementary material for: Specificity and Mechanism of Coronavirus, Rotavirus, and Mammalian Two-Histidine Phosphoesterases That Antagonize Antiviral Innate Immunity
Source: mBio. 2021 Aug 10;12(4):e01781-21. doi: 10.1128/mBio.01781-21 (PMC8406329; doi:10.1128/mBio.01781-21)
Supplement: FIG S6 [file mbio.01781-21-sf006.pdf]

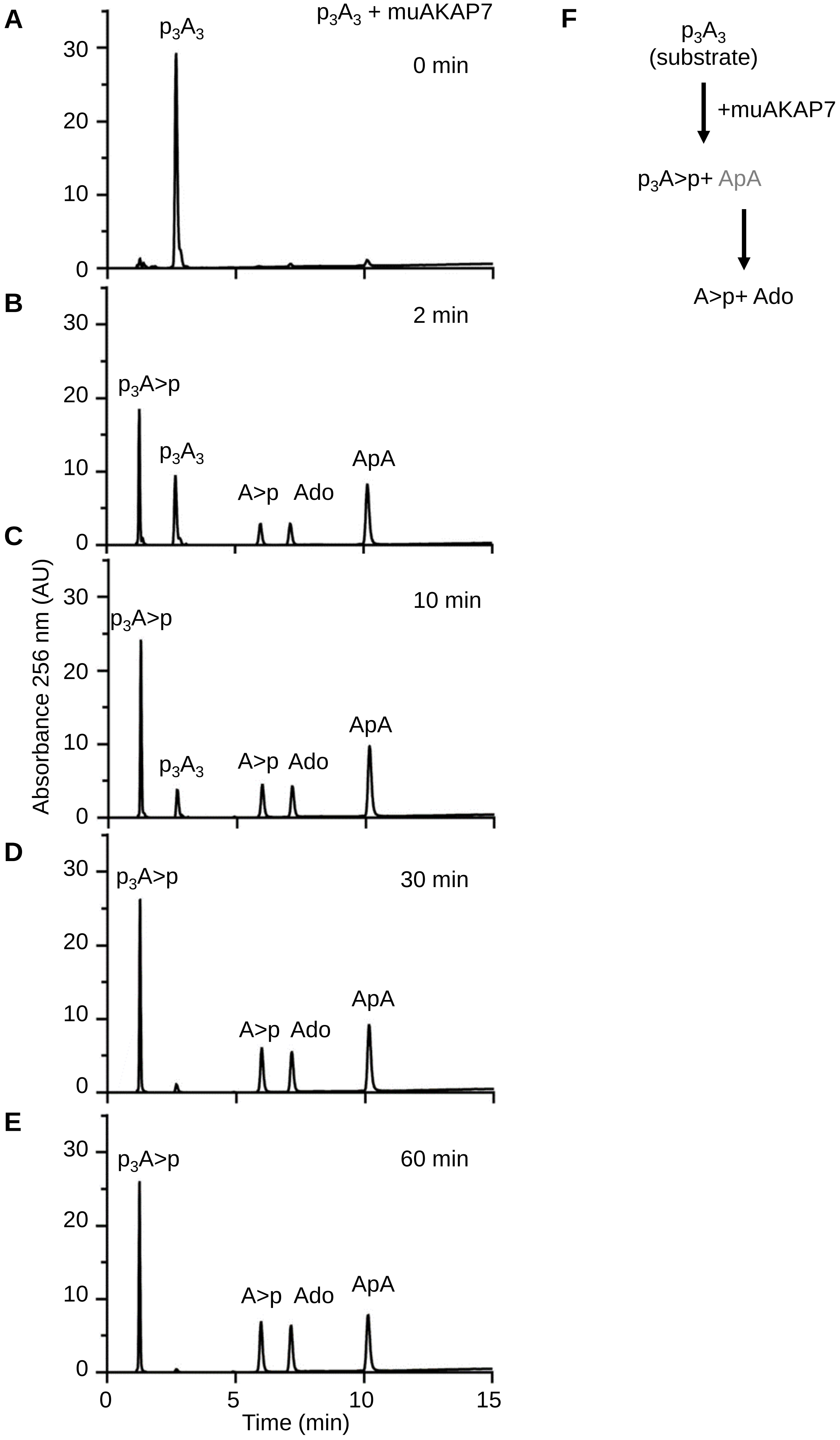

A
B
C
D
E
F
p3A3 + muAKAP7
0 min
2 min
10 min
30 min
60 min
p3A3
p3A>p
A>p
Ado
ApA
Absorbance 256 nm (AU)
Time (min)
0
5
10
15
20
30
p3A3
(substrate)
+muAKAP7
p3A>p+ ApA
A>p+ Ado

Figure S6. Time-course of 2',5'-p_3A_3 cleavage by muAKAP7. Purified 2',5'-p_3A_3 (200 µM) was incubated with muAKAP7 (1 µM) at 30°C. Samples were collected at (A) 0 min, (B) 2 min, (C) 10 min, (D) 30 min and (E) 60 min and analyzed by HPLC. The peaks were identified by comparing the elution time of known standards. The percent of substrate or products at indicated times were determined by calculating the area under the peaks on the HPLC chromatograms. (F) Schematics showing cleavage of 2',5'-p_3A_3 by muAKAP7. Reaction intermediate is shown in grey color.
